# Generative AI for rapid diffusion MRI with improved image quality, reliability, and generalizability

**DOI:** 10.1162/imag_a_00193

**Published:** 2024-06-13

**Authors:** Amir Sadikov, Xinlei Pan, Hannah Choi, Lanya T. Cai, Pratik Mukherjee

**Affiliations:** Radiology and Biomedical Imaging, University of California, San Francisco, CA, United States; Graduate Group in Bioengineering, University of California, San Francisco, CA, United States; University of California, Berkeley, CA, United States

**Keywords:** supervised learning, neural networks, MR-diffusion tensor imaging, MR-diffusion weighted imaging, brain/brain stem

## Abstract

We use generative AI to enable rapid diffusion MRI (dMRI) with high fidelity, reproducibility, and generalizability across clinical and research settings. We employ a Swin UNEt Transformers (SWIN) model, trained on Human Connectome Project (HCP) data (n = 1021) and conditioned on registered T1 scans, to perform generalized dMRI denoising. We also qualitatively demonstrate super-resolution with artificially downsampled HCP data. Remarkably, SWIN can be fine-tuned for an out-of-domain dataset with a single example scan, as we demonstrate on dMRI of children with neurodevelopmental disorders (n = 40), adults with acute traumatic brain injury (n = 40), and adolescents with intracerebral hemorrhage due to vascular malformations undergoing resection (n = 8), each cohort scanned on different scanner models with different imaging protocols at different sites. This robustness to scan acquisition parameters, patient populations, scanner types, and sites eliminates the advantages of self-supervised methods over our fully supervised generative AI approach. We exceed current state-of-the-art denoising methods in accuracy and test–retest reliability of rapid diffusion tensor imaging (DTI) requiring only 90 seconds of scan time. SWIN denoising also achieves dramatic improvements over the state-of-the-art for test–retest reliability of intracellular volume fraction and free water fraction measurements and can remove heavy-tail noise, improving biophysical modeling fidelity. SWIN enables rapid diffusion MRI with unprecedented accuracy and reliability, especially at high diffusion weighting for probing biological tissues at microscopic spatial scales. The code and model are publicly available athttps://github.com/ucsfncl/dmri-swin.

## 1 Introduction

Since its introduction in 1985, diffusion MRI (dMRI) has seen widespread adoption. The number of publications on diffusion MRI has exploded from a few hundred per year in the 1990s to tens of thousands in recent years driven by rapidly expanding scientific and clinical applications, especially to neuroscience. DMRI can provide valuable clinical information and assess tissue microstructure; however, its low signal-to-noise ratio (SNR) can limit its diagnostic and quantitative accuracy (Jones, 2012). To improve SNR, most dMRI protocols require low angular and spatial resolution or a long scan time, which limits usage in many important clinical settings. Therefore, there is great interest in having short patient scan times without compromising SNR or spatial and angular resolution.

Supervised methods have been proposed to denoise brain dMRI scans, but they are limited by their lack of generalizability to different b-values, diffusion-encoding directions, signal representations or models, scanners, and/or patient populations. They are typically trained and validated on the same dataset, such as the Human Connectome Project (HCP) (Karimi & Gholipour, 2022;Tian et al., 2020). Therefore, unsupervised/self-supervised denoising methods are preferred (Fadnavis et al., 2020), yet these methods lack the performance of supervised techniques within the trained domain and can perform variably on out-of-domain (OOD) datasets.

Most dMRI denoising methods are evaluated qualitatively or by denoising a subset of the data and evaluating accuracy compared with the full dataset. In this paper, we also evaluate denoising using external validation via test–retest reliability for Diffusion Tensor Imaging (DTI) (Mukherjee et al., 2008) and Neurite Orientation Dispersion and Density Imaging (NODDI) (Zhang et al., 2012) metrics to ensure precision and via known Structural Covariance Networks (SCNs) (Li et al., 2012;Wahl et al., 2010) of those metrics in white matter (WM) and gray matter (GM) to ensure biological accuracy. Finally, we emphasize removing heavy-tail noise, which can lead to biased biophysical metrics.

We propose to use a Swin UNEt TRansformer (SWIN) model (Hatamizadeh et al., 2022) to denoise dMRI data conditioned on registered T1 scans. Unlike other supervised methods, which train with a small subset of HCP data (Karimi & Gholipour, 2022;Tian et al., 2020), we use the full HCP dataset, training with all b-values and diffusion-encoding directions, including data augmentations: random flipping, rotation, scaling, and k-space downsampling. Training on close to 300,000 3D volumes across 1021 subjects allows SWIN to learn a denoising function that generalizes well. We also train a UNet convolutional neural network without a transformer component to determine whether network architecture affects denoising performance. We validate our approach on a held-out HCP Retest dataset and three external datasets acquired in different patient populations using different scanners and dMRI protocols, outperforming state-of-the-art unsupervised/self-supervised methods in accuracy and repeatability. Fine-tuning SWIN on even a single subject improves performance on out-of-domain datasets. Finally, our approach can also super-resolve dMRI data from HCP.

## 2 Materials and Methods

### 2.1 Data

Experiments utilized four datasets, including one of normal young adult volunteers separated into a training dataset and held-out dataset. The other three are OOD patient datasets for generalizability and clinical applicability. All datasets were acquired with the written informed consent of the participants or their legal guardians. Only deidentified data were used.

— HCP: Young adults (Essen et al., 2013) scanned with 90 diffusion-encoding directions at b-values of b = 1000, 2000, 3000 s/mm^2^with 1.25 mm resolution. We used 1021 subjects for training and held out 44 subjects in the HCP Retest dataset.
— TBI: 45 adult mild traumatic brain injury patients acquired two decades ago (Kuceyeski et al., 2019;Wahl et al., 2010) on a 3T GE scanner with 55 diffusion-encoding directions at b = 1000 s/mm^2^and 1.8 mm voxels (0.9 mm in xy after zero-interpolation in k-space). We randomly selected 5 subjects for fine-tuning and 40 subjects for testing.
— SPIN: 45 children ages 8-12 years with neurodevelopmental disorders acquired on a 3T Siemens Prisma scanner with 64 and 96 diffusion-encoding directions atb=1000,2500s/mm^2^, respectively, with 2.00 mm voxels (Mark et al., 2023). We randomly selected 5 subjects for fine-tuning and 40 subjects for testing.
— AHA: 8 children ages 8-18 years with hemorrhagic vascular malformations, primarily arteriovenous malformations (AVMs) acquired on a 3T GE MR750 with 55 diffusion-encoding directions at b = 2000 s/mm^2^and 2.00 mm voxels (zero-interpolated in-plane to 1.00 mm) over three sessions: prior to resection, 6 months postsurgery, and 1 year postsurgery (one subject had only two sessions). A ninth subject was used for fine-tuning.

For preprocessing, dMRI data were skull-stripped with Synthstrip (Hoopes et al., 2022), corrected for eddy current-induced distortions and subject movements with Eddy (Andersson & Sotiropoulos, 2016), and aligned to structural 3D T1 scans with Boundary-Based Registration (Greve & Fischl, 2009). T1 scans were skull-stripped using Synthstrip and segmented using SynthSeg (Billot et al., 2022). Due to lesions impacting SynthSeg performance in AHA data, T1 scans were instead segmented using Freesurfer’s recon-all command with the SynthSeg option enabled or with recon-all-clinical if recon-all failed. Finally, coregistered T1 and dMRI scans were resampled with 5th order spline interpolation at 1.25 mm, concatenated together, and used as inputs to the model. Negative values were clipped.

### 2.2 Denoising validation

We measure denoising and scan speedup performance in fully sampled and subsampled data. We use the minimum directions for unique fits: 6 for DTI, 15 for 4th order spherical harmonic, and 28 for 6th order spherical harmonic along with 1 b = 0 s/mm^2^volume. We select the directions to minimize the condition number of the design matrix (Skare et al., 2000;Tian et al., 2022). For NODDI, we use the fully sampled HCP acquisition. We compare SWIN and UNet with five state-of-the-art unsupervised/self-supervised dMRI denoising methods: block-matching and 4D filtering (BM4D) (Maggioni et al., 2013), Marchenko-Pastur Principal Component Analysis (MPPCA) (Veraart et al., 2016), Tensor Marchenko-Pastur Principal Component Analysis (TPCA) (Olesen et al., 2023), DDM^2^(Xiang et al., 2023), and Patch2Self (P2S) (Fadnavis et al., 2020).

We measure mean absolute error (MAE) between ground truth fully sampled data and model predictions from subsampled data for DTI, including principal eigenvector (V1), fractional anisotropy (FA), axial diffusivity (AD), radial diffusivity (RD), and mean diffusivity (MD). For higher order spherical harmonics, we used the Jensen–Shannon distance (JSD) between fully sampled ground truth and model predictions, projected onto a uniformly distributed 362-direction hemisphere (Cohen-Adad et al., 2011). DTI only utilized the lowest shell (b = 1000 s/mm^2^) for multishell datasets, whereas spherical harmonic estimation used every shell. For super-resolution, dMRI data from an HCP subject were k-space downsampled by a factor of 2 and then upsampled to emulate a low resolution acquisition.

We explore clinical translation using the AHA dataset of children with intracerebral hemorrhage before and after intervention. We measure global WM and GM DTI metrics and perilesional changes in DTI microstructure over time from both subsampled and fully sampled shells. To investigate signal rectification due to low SNR, we measure the heavily attenuated free water signal in the lateral ventricles and compare the resulting intensity histograms produced by each denoising method.

We assess repeatability using HCP Retest data by measuring within-subject coefficient of variation (CoV) for DTI and NODDI across two sessions. We consider both fully sampled and subsampled data for DTI test–retest reliability, but use full multishell data for NODDI. For repeatability in WM regions, we perform tract-based spatial statistics (TBSS) using the Johns Hopkins University (JHU) atlas (Smith et al., 2006).

SCNs are derived by computing region-wise coefficients of determination among subjects. We measure mean absolute difference between first and second sessions for SCN repeatability, using Desikan–Killiany–Tourville atlas regions for cortical GM SCNs and JHU WM atlas regions for WM SCNs. We evaluate DTI SCN repeatability on subsampled b = 1000 s/mm^2^data and NODDI SCN repeatability on fully sampled multishell data. Accuracy is determined from MAE between denoised subsampled DTI SCNs and ground truth fully sampled DTI SCNs.

For both DTI estimation and microstructural repeatability validation, we test for better SWIN performance using subject-wise, one-tailed, paired*t*-tests between it and the self-supervised methods for the HCP dataset, and between SWIN model with fine-tuning on one subject and self-supervised denoising for the three OOD datasets. For test–retest reliability of regional results, we average CoV across WM and GM regions, respectively, when conducting these statistical inference tests. We set a significance level of 0.05.

### 2.3 Training and implementation

**Fig. 1.**
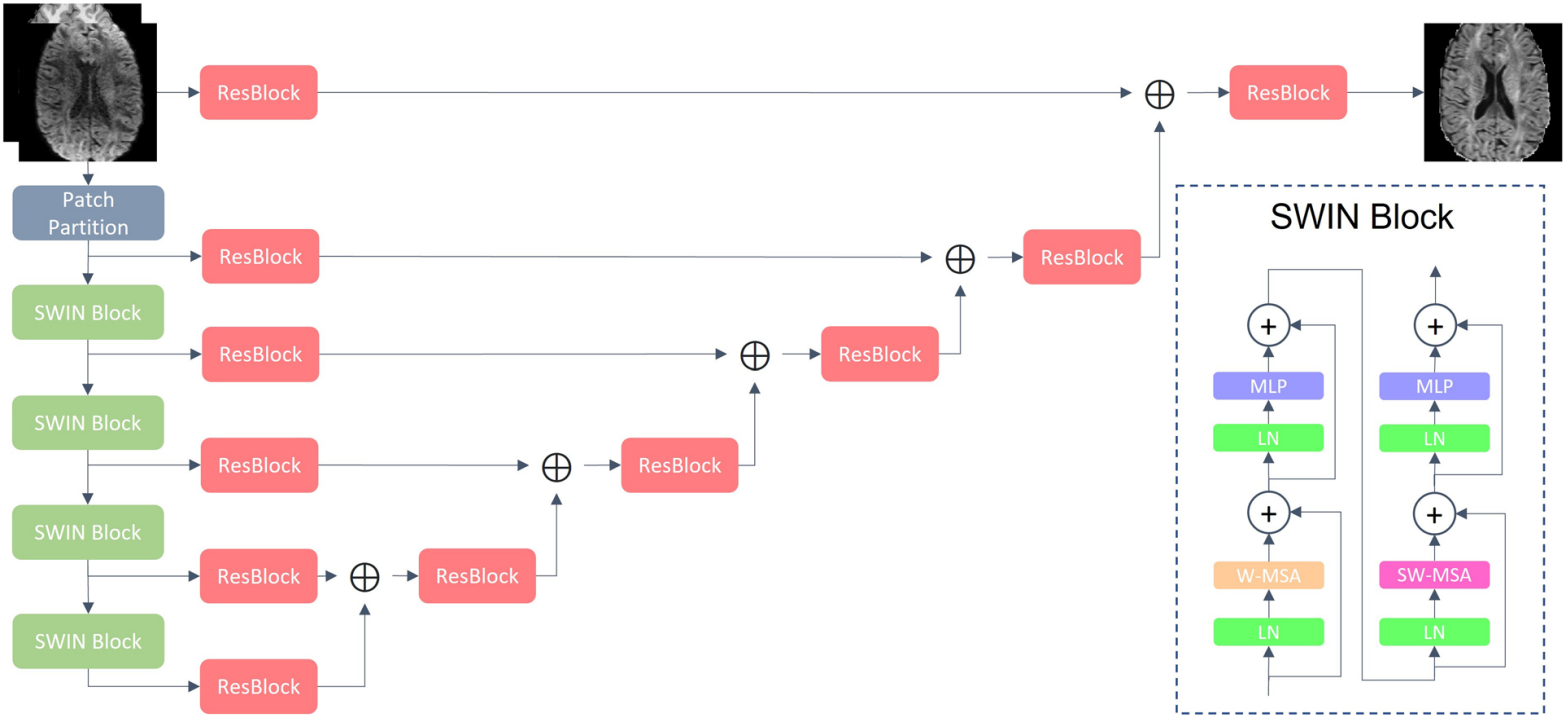
A general overview of the SWIN architecture. The input is a concatenated 3D T1 and dMRI scan, which is encoded by a Swin transformer at multiple resolutions and fed into a residual convolutional neural net (CNN) decoder, consisting of residual CNN UNet blocks (ResBlocks), to reconstruct the ground truth dMRI scan. The SWIN Block contains LayerNorm (LN), MultiLayer Perceptron (MLP), regular multihead self-attention (W-MSA), and partitioning multihead self-attention (SW-MSA) modules.

SWIN (Hatamizadeh et al., 2022;Liu et al., 2021) was implemented using PyTorch (Paszke et al., 2019) and MONAI with default model hyperparameters (Cardoso et al., 2022) (Fig. 1) and trained on an NVIDIA V100 GPU using mean-squared error loss between model output and ground truth. Ground truth was obtained from a 6th order spherical harmonic fit for each shell projected onto the acquired directions. AdamW (Loshchilov & Hutter, 2017) optimization was conducted with a learning rate of 1e-5, gradient clipping with a maximal norm of 1.0, and 16-bit precision for 14 epochs. During training, we first downsampled the dMRI scan with a probability of 0.5 in frequency space to an anisotropic resolution between 1.25 and 3 mm and linearly upsampled back to 1.25 mm resolution (Lyu et al., 2019). Patches of 128 x 128 x 128 were randomly cropped, flipped, and rotated in 90° increments along all axes. The input dMRI patch was standardized to unit variance and the input T1 patch was standardized to a standard deviation uniformly log-scaled between 0.25 and 4.0. For inference, we use a sliding window with an overlap of 0.875. Fine-tuning was achieved via additional training on external data from one held-out subject out of five with a learning rate of 1e-6 for three epochs, reporting the average result for validation. Since training and evaluation were on 1.25-mm HCP data, model predictions were 5th order spline interpolated to native dMRI resolution for external OOD dataset validation. The UNet (Kerfoot et al., 2019) was implemented using Pytorch and Monai with default model hyperparameters. UNet training, fine-tuning, and validation were conducted identically, except training continued for 20 epochs until training loss stabilized.

## 3 Results

### 3.1 Denoising performance

**Fig. 2.**
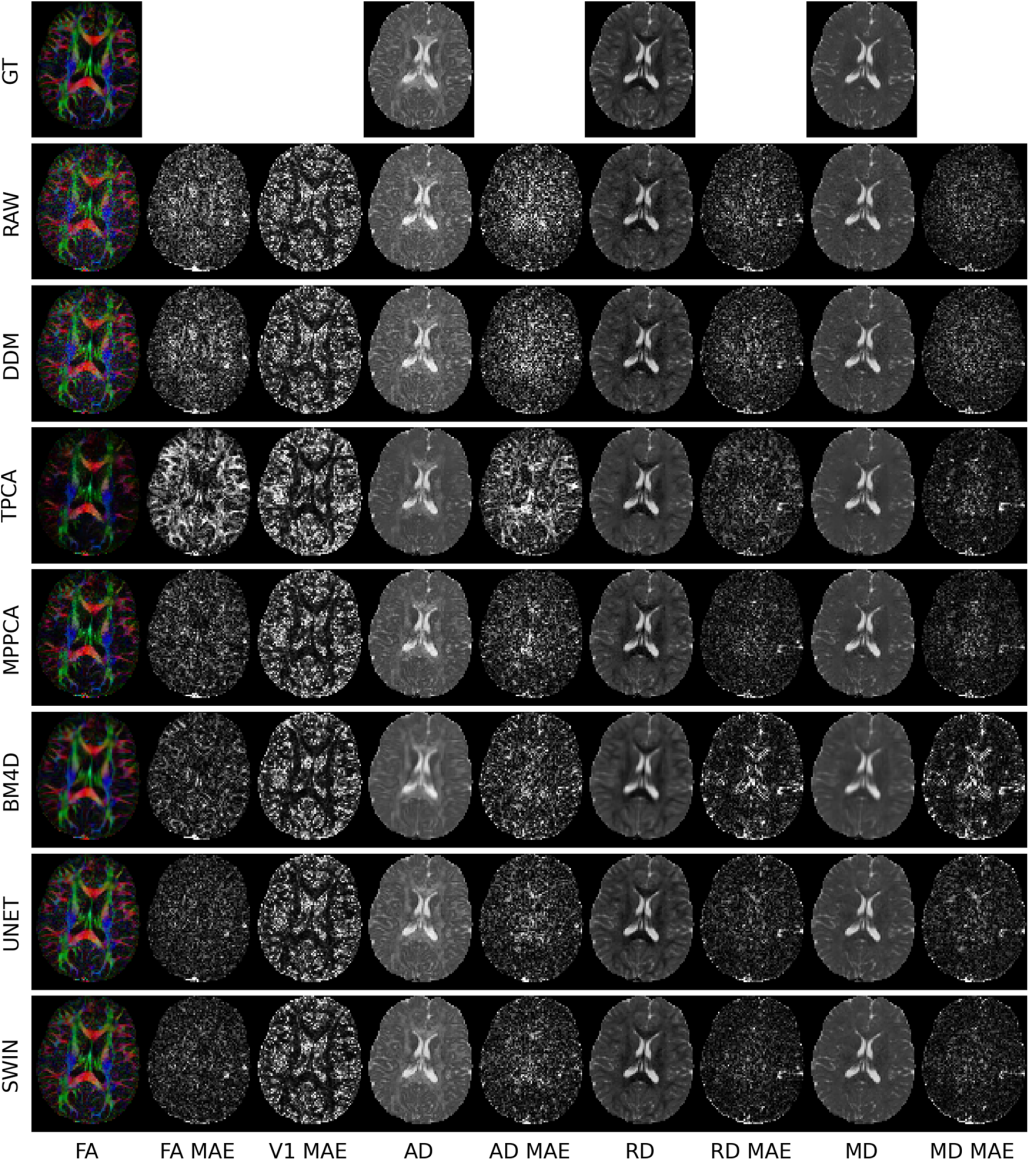
Visual comparison between the ground truth (GT), no denoising (RAW), DDM^2^, TPCA, MPPCA, BM4D, Unet, and SWIN without fine-tuning (SWIN) for denoising on validation data from the SPIN dataset. The ground truth is based on the full set of gradient-encoding directions at b = 1000 s/mm^2^whereas the RAW, DDM^2^, TPCA, BM4D, MPPCA, Unet, and SWIN images are derived from the six-direction subset. The mean absolute error (MAE) maps for each parameter are displayed to demonstrate the accuracy of each method. Lower MAE values signify higher accuracy and MAE maps that display anatomical structure could indicate denoising biases.

The SWIN model achieves the lowest MAE for almost all DTI metrics in WM and GM in the HCP and TBI validation datasets, even without any fine-tuning (Table 1). For the SPIN dataset, the SWIN model also outperforms all other models, except for FA in GM where BM4D did best. In AHA data, excepting MD and RD, SWIN achieves the lowest MAE. While UNet exceeds BM4D, MPPCA, and P2S in most settings, its performance still lags behind the SWIN model, especially after fine-tuning. Patch2Self does worse than other denoising methods, especially for V1 estimation. We include quantitative comparison with DDM^2^with one example subject from each dataset (SupplementaryTable S6). Qualitative comparisons reinforce these quantitative results. SWIN captures finer features in WM and GM microstructure without the excessive smoothing of BM4D, TPCA, and MPPCA (Figs. 2and3; SupplementaryFigs. S1 and S2). We examine the noise distribution of each denoising algorithm in the lateral ventricles of one AHA subject (Fig. 4B). Unlike other denoising algorithms, SWIN is able to transform the original data from a heavy-tailed Rician-like distribution into a more Gaussian-like distribution with significantly smaller variance and skew but greater kurtosis.

**Table 1.** The Mean Absolute Error (MAE) of FA, MD, RD, AD, and V1 estimation using six-direction HCP, SPIN, TBI, and AHA data in white matter (WM) and gray matter (GM) via no denoising (RAW), P2S, TPCA, MPPCA, BM4D, UNET, SWIN with no fine-tuning (SWIN), UNET with fine-tuning on one subject (UNET-F1), and SWIN with fine-tuning on one subject (SWIN-F1).

Dataset	Tissue	Metric	RAW	P2S	TPCA	MPPCA	BM4D	UNET	SWIN	UNET-F1	SWIN-F1	p-Value
HCP	WM	AD	0.13	0.285	0.126	0.101	0.0848	0.0913	**0.0811**			**2.5e-08**
		FA	0.0935	0.249	0.0994	0.0672	0.0563	0.0556	**0.0496**			**2.2e-19**
		MD	0.0657	0.0695	0.0497	0.0556	0.0483	0.0526	**0.0452**			**7.8e-08**
		RD	0.0773	0.124	0.0635	0.0622	0.0541	0.0558	**0.0488**			**1.0e-13**
		V1	19.7	66.9	15.9	15.2	13.9	13.4	**12.6**			**2.4e-29**
	GM	AD	0.133	0.107	0.0873	0.0903	0.0931	0.0957	**0.0794**			**3.1e-12**
		FA	0.111	0.0793	0.0559	0.0542	0.0588	0.0569	**0.0484**			**8.53-14**
		MD	0.0723	0.0728	0.0655	0.07	0.0683	0.0725	**0.0597**			**8.8e-11**
		RD	0.0849	0.0814	0.0695	0.0731	0.0719	0.0754	**0.0626**			**2.0e-12**
		V1	33.6	64.1	30.1	28.7	28.1	28.4	**26.7**			**4.6e-21**
TBI	WM	AD	0.211	0.295	0.148	0.173	0.206	0.133	0.131	0.14	**0.13**	**1.8e-24**
		FA	0.145	0.229	0.0986	0.117	0.14	0.0866	0.0922	0.0864	**0.0808**	**3.5e-25**
		MD	0.0941	0.0966	0.0767	0.0886	0.0907	0.0743	0.073	0.0784	**0.0702**	**5.3e-16**
		RD	0.116	0.138	0.0865	0.103	0.111	0.0841	0.085	0.0848	**0.076**	**3.5e-21**
		V1	26.6	67.1	23.3	24.2	26.1	21.5	22.1	21.8	**21.0**	**7.0e-29**
	GM	AD	0.218	0.136	0.135	0.173	0.217	0.147	0.149	0.151	**0.127**	**4.6e-04**
		FA	0.181	0.0966	0.0955	0.139	0.175	0.102	0.123	0.102	**0.0949**	2.5e-01
		MD	0.0908	0.0897	0.0889	0.089	0.0893	0.0905	**0.0809**	0.0926	0.0817	**3.9e-08**
		RD	0.124	0.0982	0.0983	0.11	0.12	0.102	0.102	0.104	**0.0948**	**4.8e-02**
		V1	38.0	62.0	39.1	36.6	37.6	35.5	36.2	35.6	**35.0**	**4.5e-22**
SPIN	WM	AD	0.112	0.594	0.164	0.099	0.0999	0.0869	0.0825	0.0844	**0.0767**	**4.6e-02**
		FA	0.0829	0.178	0.154	0.0768	0.0747	0.0609	0.057	0.0578	**0.0518**	**3.8e-12**
		MD	0.0553	0.203	0.0428	0.0446	0.0438	0.0491	0.044	0.0415	**0.0402**	**4.1e-02**
		RD	0.0657	0.128	0.0765	0.0561	0.0536	0.056	0.0504	0.0479	**0.0457**	**1.4e-09**
		V1	18.4	63.9	20.9	16.9	15.0	14.8	14.5	14.6	**13.8**	**1.3e-23**
	GM	AD	0.106	0.652	0.0903	0.0759	0.0823	0.0905	0.0859	0.0746	**0.0694**	**2.8e-03**
		FA	0.0994	0.193	0.0794	0.0571	**0.0508**	0.0617	0.0636	0.0559	0.0517	9.9e-01
		MD	0.0527	0.207	0.0515	0.0506	0.0652	0.0611	0.056	0.0494	**0.0471**	**4.3e-03**
		RD	0.066	0.106	0.0621	0.057	0.0703	0.0642	0.0609	0.0555	**0.0529**	**5.6e-05**
		V1	30.9	59.7	38.5	30.5	28.8	28.3	28.3	27.8	**26.9**	**4.3e-23**
AHA	WM	AD	0.145	0.337	0.11	0.0981	0.14	0.091	**0.088**	0.0987	0.0921	**7.1e-09**
		FA	0.0824	0.225	0.0854	0.0661	0.0796	0.06	0.0607	0.0736	**0.0591**	**2.7e-17**
		MD	0.0407	0.15	0.0323	**0.0312**	0.0389	0.0378	0.0371	0.0396	0.0362	1.0e+00
		RD	0.0489	0.189	0.0414	0.0378	0.0466	0.041	0.0414	0.0502	**0.0374**	1.3e-01
		V1	20.1	69.2	18.5	18.1	19.4	16.6	17.2	16.8	**16.0**	**5.0e-25**
	GM	AD	0.173	0.462	0.0855	0.101	0.161	0.0866	0.0943	0.0917	**0.0801**	**1.1e-08**
		FA	0.0988	0.107	0.0625	0.0685	0.0947	0.0569	0.0721	0.0577	**0.0535**	**7.1e-10**
		MD	0.0484	0.247	0.0382	**0.0375**	0.0449	0.047	0.0445	0.057	0.0441	1.0e+00
		RD	0.0611	0.183	0.0407	**0.0454**	0.0574	0.0497	0.0516	0.0588	0.0464	1.0e+00
		V1	31.1	64.7	32.3	29.4	30.5	28.5	29.7	28.3	**27.7**	**1.8e-25**

We include the maximum p-value from subject-wise, paired, one-tailed*t*-tests between the self-supervised models and the SWIN model for the HCP dataset and the SWIN-F1 model for the OOD datasets. Best MAE results and significant p-values (p < 0.05) are bolded.

**Fig. 3.**
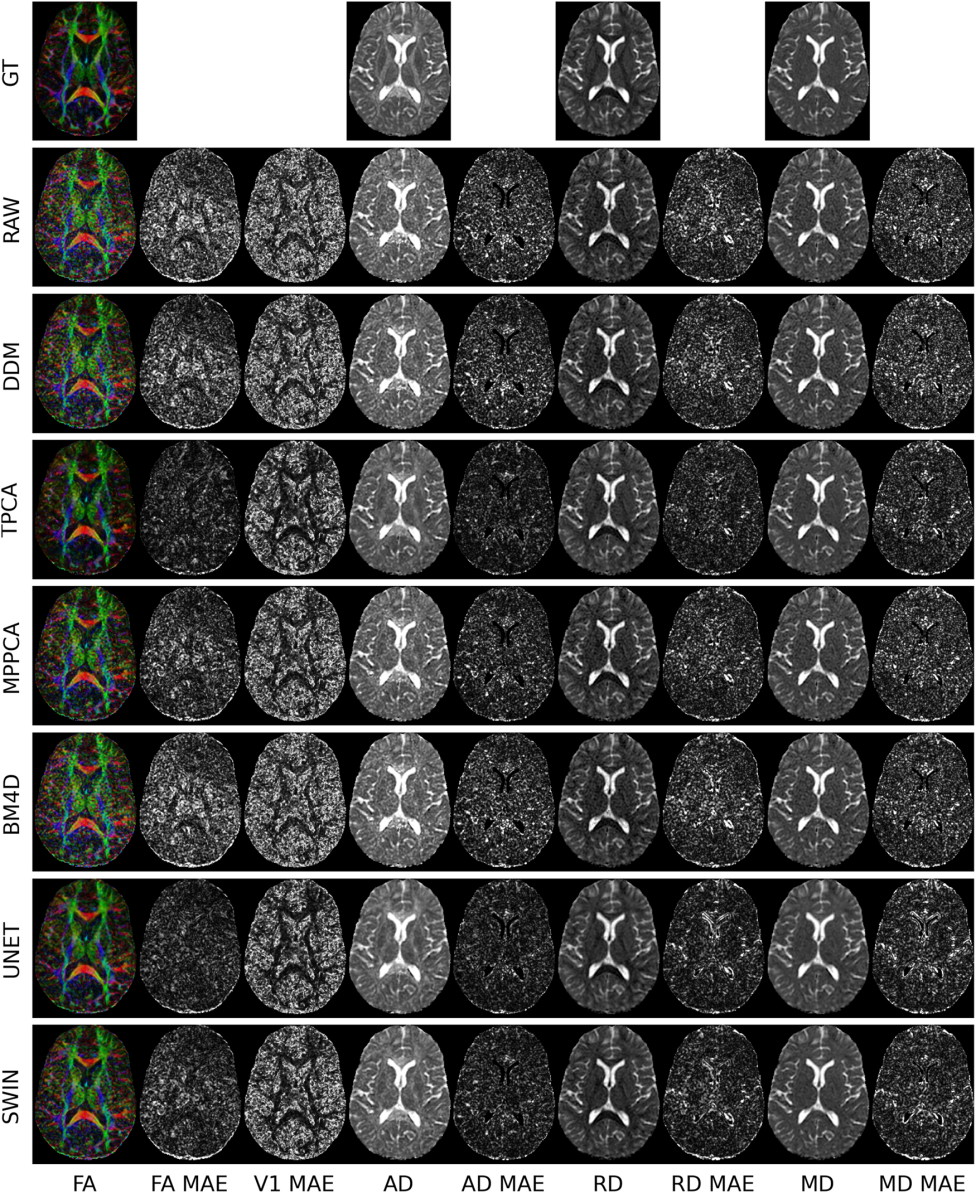
Visual comparison between the ground truth (GT), no denoising (RAW), DDM^2^, TPCA, BM4D, MPPCA, UNET, and SWIN without fine-tuning (SWIN) for denoising on validation data from the TBI dataset. The ground truth is based on the full set of gradient-encoding directions at b = 1000 s/mm^2^whereas the RAW, DDM^2^, TPCA, BM4D, MPPCA, Unet, and SWIN images are derived from the six-direction subset. The mean absolute error (MAE) maps for each parameter are displayed to demonstrate the accuracy of each method. Lower MAE values signify higher accuracy and MAE maps that display anatomical structure could indicate denoising biases.

**Fig. 4.**
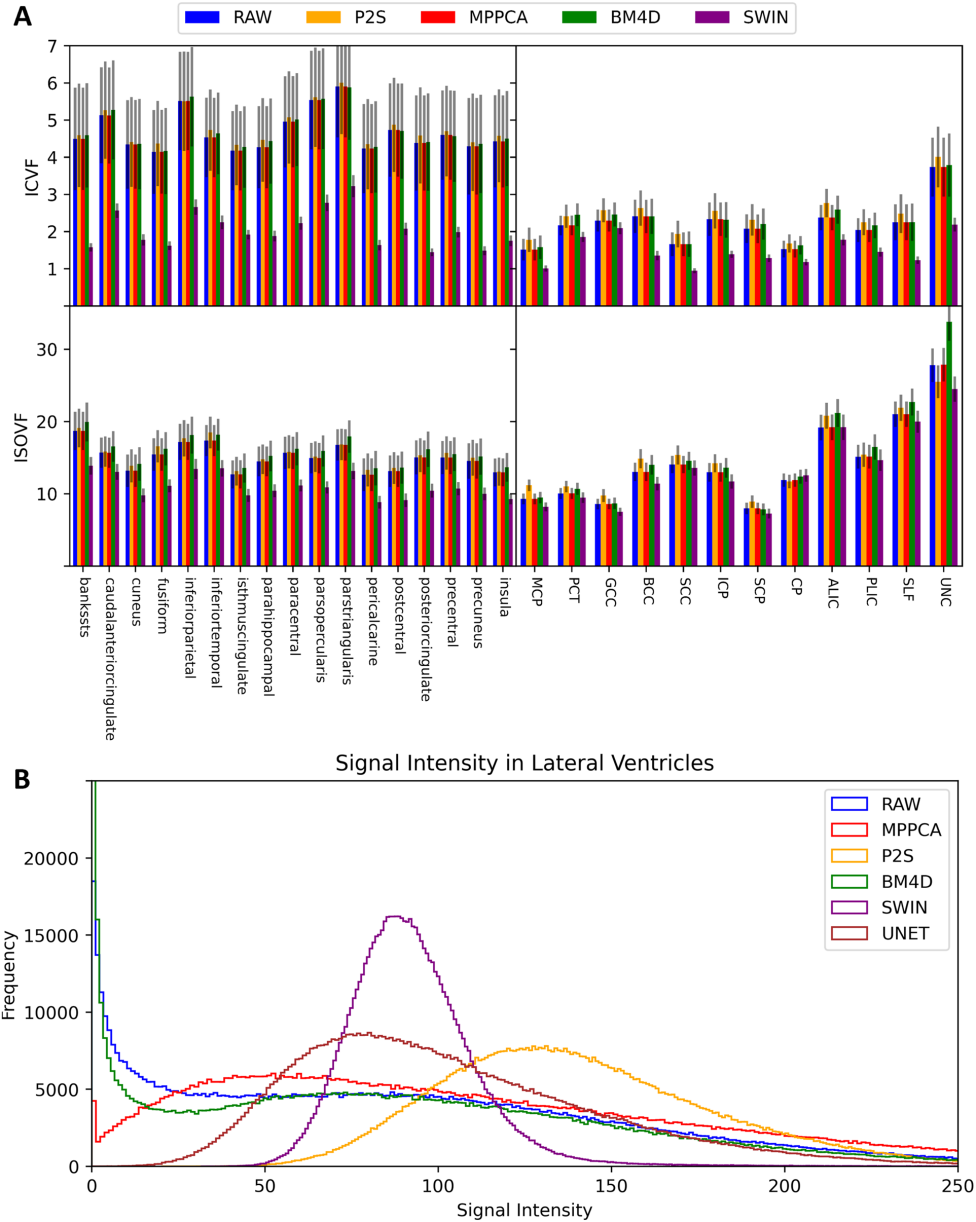
(A) ICVF and ISOVF CoV (%) in select WM and GM regions and (B) histogram of signal intensity in the b = 2000 s/mm^2^shell in the lateral ventricles of an AHA patient. Results using no denoising (RAW), P2S, BM4D, MPPCA, SWIN, and UNET denoising are displayed.

### 3.2 Test–retest reliability

SWIN achieves the lowest CoV between test and retest datasets for all metrics in GM and AD and MD in WM using the 6-direction subsampled shell, as well as MD and RD in both GM and WM for the 90-direction fully sampled shell (Table 2). For 90 directions, applying no denoising yields the lowest CoV for AD, FA, and MD in WM and AD in GM, while MPPCA does best for FA in GM. We include region-wise DTI CoV values for the six-direction subsampled shell in GM (SupplementaryTables S9, S10, S11, and S12) and WM (SupplementaryTables S13, S14, S15, and S16).

**Table 2.** Average CoV (%) across all WM and GM regions for DTI estimation using 6- and 90-direction subsets of the B1000 shell and NODDI estimation using all available data.

Validation	Tissue	Metric	RAW	P2S	MPPCA	BM4D	SWIN	p-Value
6-Direction DTI CoV	WM	AD	2.438	3.02	2.526	2.43	**2.339**	**1.4e-05**
		FA	**2.603**	7.492	3.09	2.912	2.922	1.0e+00
		MD	2.986	3.429	3.014	2.987	**2.901**	**1.5e-04**
		RD	5.273	**4.009**	5.136	5.123	4.846	1.0e+00
	GM	AD	2.447	2.451	2.54	2.436	**2.295**	**2.6e-04**
		FA	4.365	5.782	6.267	4.5	**4.186**	1.3e-01
		MD	2.518	2.529	2.536	2.524	**2.436**	**3.5e-04**
		RD	2.788	2.615	2.73	2.715	**2.611**	4.5e-01
90-Direction DTI CoV	WM	AD	**1.746**	1.974	1.781	1.783	1.826	1.0e+00
		FA	**1.208**	1.313	1.209	1.272	1.304	1.0e+00
		MD	**1.849**	2.087	1.873	1.851	1.853	6.3e-01
		RD	2.819	2.97	2.831	2.749	**2.612**	**4.8e-04**
	GM	AD	**1.579**	1.703	1.582	1.613	1.628	1.0e+00
		FA	2.614	2.651	**2.557**	3.004	3.167	1.0e+00
		MD	1.624	1.748	1.627	1.613	**1.602**	1.1e-01
		RD	1.694	1.809	1.698	1.659	**1.627**	**6.1e-03**
NODDI CoV	WM	ICVF	2.585	2.854	2.584	2.676	**1.68**	**9.0e-22**
		ODI	5.349	6.93	5.389	5.617	**5.042**	**1.3e-02**
		ISOVF	15.54	15.96	15.54	16.74	**14.41**	**9.2e-09**
	GM	ICVF	4.943	5.068	4.943	5.012	**2.487**	**2.2e-54**
		ODI	1.776	**1.77**	1.78	1.982	1.792	8.7e-01
		ISOVF	14.9	15.28	14.9	15.65	**11.13**	**2.1e-43**
6-Direction DTI SCN MAE	WM	AD	0.3707	0.3186	**0.3164**	0.3494	0.3273	
		FA	0.2163	0.1815	0.1643	0.1879	**0.1544**	
		MD	0.2615	0.2691	0.2628	0.2632	**0.2478**	
		RD	0.1703	0.2034	0.1646	0.1689	**0.1593**	
	GM	AD	0.182	**0.159**	0.1654	0.1672	0.1613	
		FA	0.2276	0.3128	0.2373	0.2104	**0.1763**	
		MD	0.1399	**0.1356**	0.1381	0.138	0.136	
		RD	0.127	0.1269	0.1306	0.13	**0.1265**	
SCN Repeatability Error	WM	AD	0.1413	0.1479	**0.1295**	0.1384	0.134	
		FA	0.1299	0.1231	**0.1203**	0.1247	0.1207	
		MD	0.1386	0.1428	0.1387	0.1415	**0.1378**	
		RD	0.1351	0.137	**0.1309**	0.1354	0.133	
		ICVF	0.2528	0.1891	0.2524	0.2759	**0.06437**	
		ODI	0.1364	**0.05801**	0.1356	0.1388	0.07944	
		ISOVF	0.3568	0.3329	0.3568	0.3689	**0.1298**	
	GM	AD	0.1547	**0.1314**	0.1737	0.1541	0.1494	
		FA	0.1491	**0.1062**	0.2125	0.178	0.1636	
		MD	0.1296	0.1275	0.1293	0.1272	**0.1271**	
		RD	0.1253	0.1259	0.1239	0.1245	**0.1229**	
		ICVF	0.5988	0.565	0.5987	0.6192	**0.1017**	
		ODI	0.165	0.1688	0.1683	0.1652	**0.1142**	
		ISOVF	0.5177	0.4929	0.5177	0.5255	**0.1196**	

We include the maximum p-value from subject-wise, paired, one-tailed*t*-tests between the self-supervised models and the SWIN model for DTI and NODDI CoV. Significant p-values (p < 0.05) are bolded. The Mean Absolute Error (MAE) for DTI SCN estimation using six-direction subsets and the repeatability error for DTI and NODDI SCNs using all available data. Results using no denoising (RAW), P2S, BM4D, MPPCA, and SWIN denoising are displayed. Best results are bolded.

For repeatability of intracellular volume fraction (ICVF), fiber orientation dispersion index (ODI), and free water fraction (ISOVF), where all acquired data were used, SWIN outperforms all other denoising methods except for ODI estimation in GM, where P2S is slightly better. In particular, SWIN excels at ICVF repeatability, achieving close to 50% lower CoV than the next best method on average and achieves dramatically lower CoV on regional GM and WM measurements (Fig. 4A). SWIN also achieves considerably better test–retest reliability than the other denoising approaches for ISOVF as well, especially in global and regional GM measurements (Fig. 4A). We include region-wise NODDI CoV values in GM (SupplementaryTables S17, S18, and S19) and WM (SupplementaryTables S20, S21, and S22).

SWIN generates the most accurate FA, MD, and RD SCNs in WM and FA and RD SCNs in GM. Patch2Self achieves the lowest error for AD and MD SCN in GM, while MPPCA has the most accurate AD SCN in WM. MPPCA yields the lowest SCN repeatability error in WM for AD, FA, and RD, while Patch2Self has the most repeatable SCN in WM for ODI and GM for AD and FA. SWIN has the lowest SCN repeatability error for MD, ICVF, and ISOVF in WM plus MD, RD, ICVF, ODI, and ISOVF in GM. SWIN performed remarkably better than all competing methods for ICVF and ISOVF, achieving CoV values one-sixth to one-third of those from no denoising or denoising with P2S, MPPCA, or BM4D.

### 3.3 Clinical validation

**Fig. 5.**
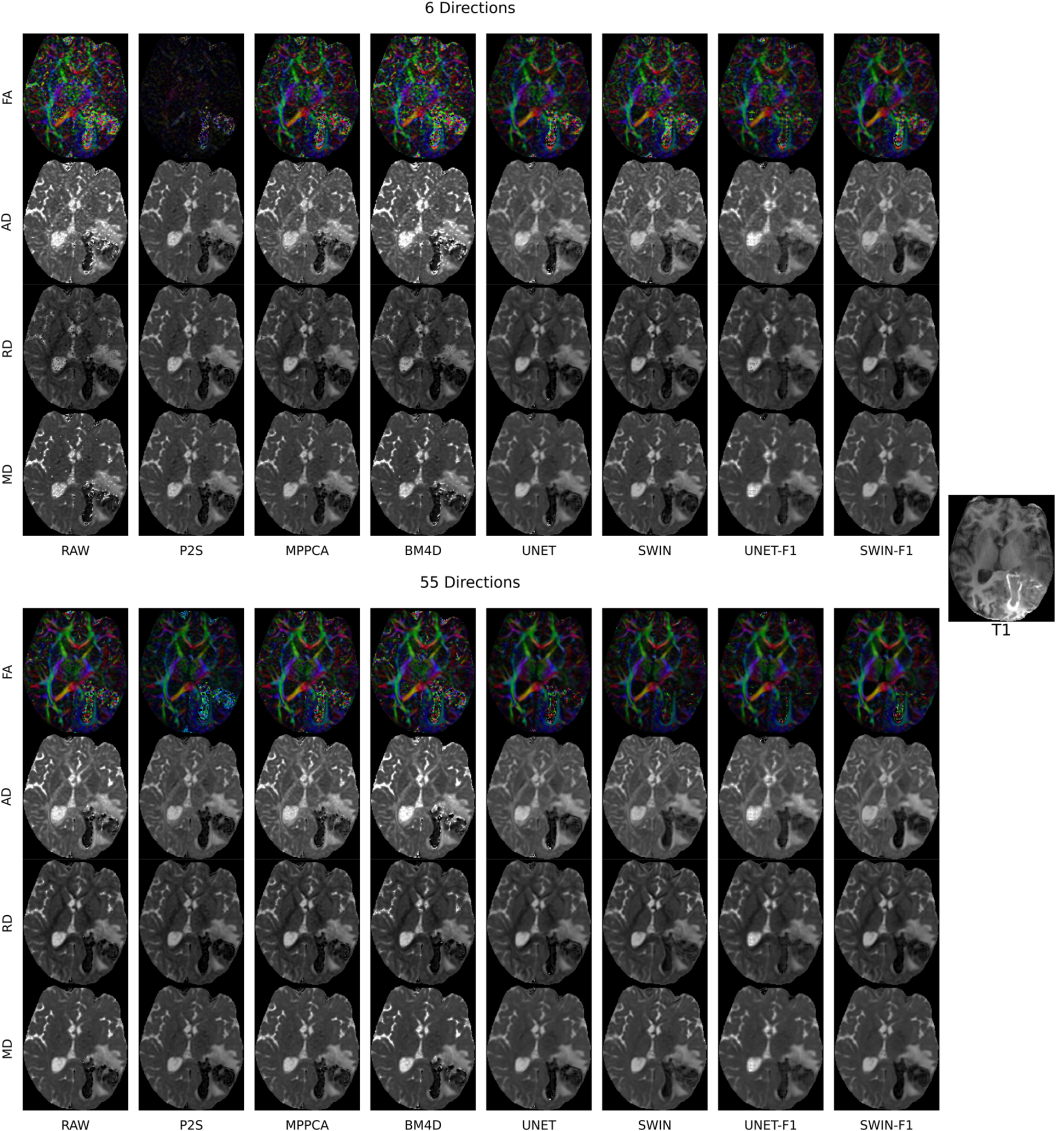
Effect of denoising on a 6-direction subset as well as a full shell (55 directions) for a session with poor quality data: contrast-to-noise ratio (CNR) = 1.2375 measured by Eddy in FSL. DTI metrics derived from no denoising (RAW), Patch2Self (P2S), MPPCA, BM4D, UNet denoising without fine-tuning (UNET), and SWIN denoising without fine-tuning (SWIN), UNet denoising with fine-tuning (UNET-F1), and SWIN denoising with fine-tuning (SWIN-F1) are displayed as well as the T1 anatomical image. The subject has a left temporal hemorrhage due to an arteriovenous malformation, as shown on the T1 image. The SWIN model is better able to remove the lesional and perilesional noise and is more consistent with the underlying anatomy than the self-supervised methods.

SWIN achieves better denoising even in poor quality data, including the lowest CNR scan in the AHA dataset (Fig. 5). SWIN denoising with only 6 directions approaches the image quality of all 55 directions, resulting in a 9-fold speedup of scan time. Applying SWIN to all 55 directions removes much of the FA noise from the AVM and its surrounding hemorrhage. Fine-tuning leads to further improvements in image quality for both the UNet and SWIN models.

SWIN denoising with 6 directions produces DTI values consistent with those of other denoising algorithms with all 55 directions (SupplementaryFig. S3). SWIN achieves lower FA than the other methods when limited to 6 directions, barring P2S which yields unrealistically low values for WM. Hence, SWIN can reduce noise that artifactually inflates FA. Apart from FA in GM, SWIN denoising generates similar values for both 6 directions and 55 directions, indicating that microstructural metrics remain consistent, even as angular resolution is increased. SWIN also consistently produces lower FA, MD, RD, and AD values than other denoising methods in the perilesional space across all three sessions in AHA subjects (SupplementaryFig. S4). MPPCA, BM4D, and no denoising (RAW) tend to follow the same values.

### 3.4 Super-resolution

**Fig. 6.**
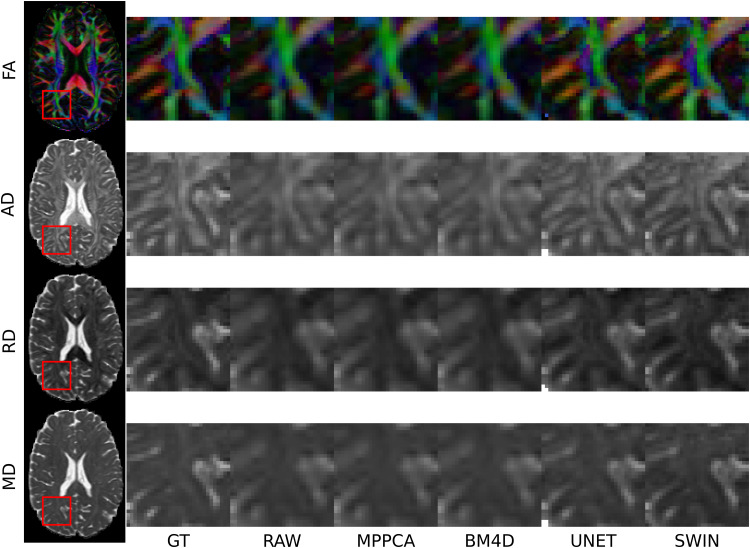
Visual comparison between the ground truth (GT), no postprocessing (RAW), MPPCA, BM4D, Unet, and SWIN without fine-tuning (SWIN) for super-resolution in the posterior periventricular WM (indicated by the red bounding box) of an HCP subject. Data were k-space downsampled by a factor of 2 and then upsampled with 5th order spline interpolation back to 1.25 mm.

Although SWIN was not trained for super-resolution, it can be used to resample a dMRI dataset to 1.25 mm resolution (Fig. 6). SWIN captures fine anatomic details in posterior periventricular WM, avoiding the excessive blurring of BM4D and MPPCA, while achieving significantly lower MAE in all DTI metrics (SupplementaryTable S5).

## 4 Discussion

To the best of our knowledge, this is the first supervised dMRI denoising method that can be applied, without modification, to dMRI from widely varying scanners, patient populations, and acquisition parameters. The novelty of our approach and our large improvement over the previous SOTA lie in the way we train our model. Instead of hard-coding diffusion-encoding directions, we trained our model to learn a denoising function that performs well in a wide range of q-space which increases its robustness to changes in SNR and contrast. By doing so, we also increased the size of our training set by a factor of 288 and made fine-tuning on even one example dMRI scan feasible. We believe that this training protocol should be feasible for other noise-limited MRI contrasts, such as fMRI, as well as other imaging modalities, such as CT. We validated our approach on held-out scans from the HCP dataset and three external OOD datasets: children with neurodevelopmental disorders, adults with TBI, and both children and adolescents with intracranial hemorrhage before and after resection of their vascular malformations. We showed that SWIN produces more accurate DTI metrics and spherical harmonic coefficients than other denoising methods with the minimum amount of data required for a unique fit. We also demonstrated the superior test–retest reliability of SWIN for both DTI and NODDI metrics as well as SCNs derived from those metrics. Finally, we showed that SWIN denoising can track brain microstructural changes with greater accuracy than other denoising models.

With SWIN denoising, drastic scan time speedup is possible. Most dMRI protocols require at least 5 b = 0 s/mm^2^and 30 b = 1000 s/mm^2^acquisitions to obtain accurate DTI metrics, taking at least 10 minutes for high spatial resolution on typical clinical MR scanners available in the community (Jones, 2004). With SWIN, accurate high-resolution DTI is achievable in under 2 minutes. Indeed, on the scanners which acquired the data, a minimal 6-direction acquisition takes 90 seconds for HCP, 100 seconds for TBI or AHA, and only 20 seconds for SPIN. This speedup enables high-resolution dMRI in uncooperative populations by mitigating motion artifacts.

SWIN showed high repeatability in all cases, especially for NODDI metrics of tissue intracellular volume fraction and free water fraction, often achieving better than 50% lower CoV than the next best method. This could reflect SWIN’s approximately Gaussian output distribution. Unlike other denoising methods, SWIN removes the heavy tail noise inherent in dMRI data. Even BM4D, which is designed to correct for Rician noise, fails at this task. SWIN and, to a lesser extent, UNet succeed partly because, unlike other denoising models, they leverage T1 anatomical information for tissue segmentation, differentiating GM from WM from CSF. Furthermore, both models are trained to reduce mean-squared error, which heavily penalizes outliers and thus reduces the heavy tail.

There were cases where SWIN was not best. For instance, SWIN underperforms MPPCA in WM for 6th order spherical harmonic fitting, even in HCP data, possibly because SWIN denoises one direction at a time, whereas MPPCA collectively denoises all directions (SupplementaryTable S7). By processing each dMRI volume separately, we consider the full brain volume and avoid artifact propagation across volumes but do not utilize correlation across volumes, such as the transformer patch-based approach inKarimi & Gholipour (2022). Due to GPU memory constraints, a trade-off exists between utilizing spatial and angular correlations. Data compression techniques, such as quantized variational autoencoders, might overcome this obstacle (Duan et al., 2023).

Our results were achieved with simple data augmentations, although using more extensive simulations, such as those inMuckley et al. (2021), could lead to greater generalizability. In addition, all inputs were resampled to 1.25 mm resolution, but with further data augmentation or resolution-independent architectures (Dehghani et al., 2023), it may be possible to perform denoising on native resolution if super-resolution is not desired. Our results require a T1 anatomical volume to be acquired in conjunction with the dMRI data, which we found improved denoising fidelity. While most datasets which have dMRI data also collect T1, this might not be always the case. Further work is needed to determine the importance of adding anatomical information to denoising performance. Finally, our denoising method is designed to be performed after correction for patient movement, susceptibility-induced distortions, and eddy-current correction via the Eddy command as well as coregistration with a T1 scan. However, it may be fruitful to pursue 2D denoising which cleans raw data slice-by-slice to improve Eddy itself and yield better downstream results.

We trained a simple UNet which, while performing better than self-supervised methods, trailed the SWIN model. This could reflect the ability of SWIN transformers to capture long-range dependencies better than UNets, especially when trained with enough data (Dosovitskiy et al., 2020). Since the same hyperparameters were used for both SWIN and UNet, it may be that the hyperparameters were simply more optimal for the SWIN model. Further hyperparameter optimization is required to determine the best architecture. In principle, we believe that most advanced neural network architectures, with sufficient complexity, would perform adequately.

Some of the generalizability of our model might be attributed to grokking, a phenomenon that occurs when a neural network with good training loss but poor generalization will, upon further training, transition to perfect generalization (Power et al., 2022). We anecdotally observed grokking to occur after the sixth epoch of training and suspect this is due to using weight decay and AdamW adaptive stochastic gradient descent training on a sufficiently large dataset with data augmentation. A better understanding of what leads to grokking could be instrumental in designing generative AI models that generalize well at scale for healthcare.

Finally, fine-tuning on even one subject consistently led to improved denoising performance for DTI, but results were mixed for higher-order spherical harmonics. This could be because fine-tuning reduces model bias, but increases variance which can accumulate error over the 15 or 28 directions used to compute the 4th or 6th order spherical harmonics, respectively, compared with the six directions used in DTI estimation. Moreover, there were no significant benefits from fine-tuning on more than one subject. Recent work has demonstrated that large language models can effectively learn from a single example, and our model, while significantly smaller, might also exhibit similar behavior via successful domain adaptation with limited fine-tuning data. To the best of our knowledge, fine-tuning has never been reported before in the context of dMRI denoising and merits further investigation.

## Supplementary Material

Supplementary Material

## Data Availability

We make the source code and model publicly available athttps://github.com/ucsfncl/dmri-swin. Of the four imaging datasets used in this report, the Human Connectome Project (Essen et al., 2013) is a widely used publicly available dataset, two others have been previously utilized for clinical research (Kuceyeski et al., 2019;Mark et al., 2023) but not for AI research, and the fourth has not been previously published.
