# Supplementary material for: Generative AI for rapid diffusion MRI with improved image quality, reliability, and generalizability
Source: Imaging Neurosci (Camb). 2024 Jun 13;2:imag-2-00193. doi: 10.1162/imag_a_00193 (PMC12272261; doi:10.1162/imag_a_00193)
Supplement: Supplementary Material [file imag_a_00193-supp.pdf]

Generative AI for Rapid Diffusion MRI with Improved Image Quality, Reliability and Generalizability Supplementary

Amir Sadikov[1,2], Xinlei Pan[3], Hannah Choi[1], Lanya T. Cai[1], and Pratik Mukherjee[1,2]

[1] Radiology and Biomedical Imaging, University of California, San Francisco
[2] Graduate Group in Bioengineering, University of California, San Francisco
[3] University of California, Berkeley
amir.sadikov@ucsf.edu

1 Introduction

For a list of all abbreviations used in the manuscript and their definitions, see Table S1.

Table S1: Table of Abbreviations

Abbreviation	Definition
dMRI	Diffusion MRI
DTI	Diffusion Tensor Imaging
NODDI	Neurite Orientation Dispersion and Density Imaging
SCN	Structural Covariance Network
HCP	Human Connectome Project
AHA	American Heart Association
TBSS	Tract-Based Spatial Statistics
JHU	Johns Hopkins University
MAE	Mean Absolute Error
AVM	Arteriovenous Malformation
JSD	Jensen–Shannon Distance
CoV	Coefficient of Variation
SNR	Signal-to-Noise Ratio
CNR	Contrast-to-Noise Ratio
OOD	Out-Of-Domain
WM	White Matter
GM	Gray Matter
GT	Ground Truth
RAW	No Denoising Applied
BM4D	Block-Matching and 4D filtering
MPPCA	Marchenko-Pastur Principal Component Analysis
P2S	Patch2Self
UNET-F1	UNET with fine-tuning on one subject
Swin UNETR/SWIN	Swin UNEt TRansformers
SWIN-F1	SWIN with fine-tuning on one subject
V1	Principal Eigenvector
FA	Fractional Anisotropy
AD	Axial Diffusivity
RD	Radial Diffusivity
MD	Mean Diffusivity
ICVF	Intracellular Volume Fraction
ODI	fiber Orientation Dispersion Index
ISOVF	Free Water Fraction
MCP	Middle Cerebellar Peduncle
PCT	Pontine Crossing Tract
GCC	Genu of Corpus Callosum
BCC	Body of Corpus callosum
SCC	Splenium of corpus callosum
FX	Fornix
CST	Corticospinal Tract

ML	Medial Lemniscus
ICP	Inferior Cerebellar Peduncle
SCP	Superior Cerebellar Peduncle
CP	Cerebral Peduncle
ALIC	Anterior Limb of Internal Capsule
PLIC	Posterior Limb of Internal Capsule
RLIC	Retrolenticular Limb of Internal Capsule
ACR	Anterior Corona Radiata
SCR	Superior Corona Radiata
PCR	Posterior Corona Radiata
PTR	Posterior Thalamic Radiation
SS	Sagittal Stratum
EC	External Capsule
CGC	Cingulum (Cingulate Gyrus)
CGH	Cingulum (Parahippocampal)
FXST	fornix and Stria Terminalis
SLF	Superior Longitudinal Fasciculus
SFO	Superior Fronto-Occipital Fasciculus
UNC	Uncinate Fasciculus
TPT	Tapetum

2 Methods

A mask for the perilesional space was found by taking taking the largest connected component of the region that Freesurfer *recon-all* segments as *"unknown"* inside the brain mask. We dilate this mask two-fold and only take the dilated portion to be perilesional.

The SWIN model architecture followed the default model hyperparameters (Table S2), while the UNET model architecture followed the following model hyperparameters (Table S3).

Table S2: The model configuration of the SWIN model

Embed Dimension	Feature Size	Number of Blocks	Window Size	Number of Heads	Parameters
768	48	[2, 2, 2, 2]	[7, 7, 7]	[3, 6, 12, 24]	62.0M

Table S3: The model configuration of the UNET model

Embed Dimension	Feature Size	Number of Blocks	Filter Size	Parameters
768	48	[2, 2, 2, 2]	[3, 3, 3]	43.2M

3 Results

For each of the denoising methods used, we have enumerated the approximate runtime for a standard DTI acquisition (i.e. 5 b=0 s/mm^2 and 30 b=1000 s/mm^2 volumes) (Table S4). The SWIN, Unet, and BM4D methods also have linear computational complexity with respect to the number of volumes, while other methods, such as MPPCA, TPCA, and P2S, have at least cubic computational complexity.

Table S4: The inference time of the denoising methods used in the paper in GPU/CPU minutes respectively.

BM4D	MPPCA	P2S	TPCA	UNET	SWIN
3 CPU hrs	0.5 CPU hrs	1 CPU hrs	0.75 CPU hours	0.3 GPU Hours	0.5 GPU hours

Table S5: MAE of FA, MD, RD, AD, and V1 estimation using six-direction HCP data downsampled by a factor of 2 in white matter (WM) and gray matter (GM) via no denoising (RAW), P2S, MPPCA, BM4D, UNET, and SWIN. We include the maximum p-value from subject-wise, paired, one-tailed t-tests between the self-supervised models and the SWIN model. Best MAE results and significant p-values (p <0.05) are **bolded**.

Tissue	Metric	RAW	P2S	MPPCA	BM4D	UNET	SWIN	P-Value
WM	AD	0.166	0.286	0.131	0.125	0.113	**0.107**	**4.1e-28**
	FA	0.096	0.241	0.0748	0.0718	0.0672	**0.0641**	**2.3e-26**
	MD	0.109	0.114	0.0853	0.0814	0.0737	**0.0707**	**9.0e-21**
	RD	0.113	0.18	0.0875	0.0837	0.0766	**0.0737**	**9.9e-22**
	V1	21.3	64.2	16.4	16.0	14.1	**13.9**	**2.5e-38**
GM	AD	0.203	0.215	0.158	0.153	0.139	**0.127**	**5.6e-27**
	FA	0.0873	0.0854	0.0652	0.065	0.0615	**0.0569**	**4.2e-25**
	MD	0.177	0.177	0.14	0.133	0.12	**0.108**	**8.3e-28**
	RD	0.178	0.186	0.14	0.134	0.12	**0.108**	**5.4e-29**
	V1	43.3	62.7	33.7	32.7	29.4	**28.5**	**8.7e-46**

Table S6: MAE of FA, MD, RD, AD, and V1 estimation using six-direction HCP, SPIN, TBI, and AHA data from one example subject in white matter (WM) and gray matter (GM) via no denoising (RAW), P2S, DDM, MPPCA, BM4D, UNET, SWIN with no finetuning (SWIN), and SWIN with finetuning on one subject (SWIN-F1). Best results are **bolded**.

Dataset	Tissue	Metric	RAW	P2S	DDM	TPCA	MPPCA	BM4D	UNET	SWIN	UNET-F1	SWIN-F1
HCP	WM	AD	0.13	0.315	0.256	0.135	0.107	0.0928	0.0927	**0.0851**		
		FA	0.0967	0.271	0.197	0.0976	0.0685	0.0619	0.055	**0.0499**		
		MD	0.0712	0.0751	0.0986	0.0569	0.0629	0.0567	0.0571	**0.0509**		
		RD	0.0833	0.123	0.15	0.0646	0.0674	0.0619	0.0587	**0.0524**		
		V1	19.7	71.3	54.5	16.4	15.4	14.6	13.5	**12.6**		
	GM	AD	0.145	0.123	0.133	0.0994	0.101	0.109	0.103	**0.0879**		
		FA	0.113	0.0894	0.072	0.0528	0.056	0.0655	0.0593	**0.0486**		
		MD	0.0858	0.0829	0.116	0.0783	0.0814	0.0812	0.0791	**0.0697**		
		RD	0.0948	0.0893	0.119	0.0795	0.0831	0.0833	0.0805	**0.0712**		
		V1	33.6	67.4	57.1	30.6	28.6	28.9	28.0	**26.5**		
TBI	WM	AD	0.218	0.298	0.191	0.151	0.172	0.214	0.132	0.131	0.135	**0.128**
		FA	0.151	0.221	0.131	0.096	0.115	0.144	0.0848	0.0912	0.0851	**0.0771**
		MD	0.0992	0.103	0.102	0.0812	0.0939	0.095	0.0779	0.0769	0.0778	**0.0723**
		RD	0.124	0.138	0.119	0.0903	0.108	0.118	0.0881	0.0891	0.0872	**0.0779**
		V1	28.0	66.1	27.0	23.9	25.1	27.4	22.4	23.4	22.7	**22.0**
	GM	AD	0.202	0.148	0.169	0.127	0.155	0.197	0.134	0.143	0.134	**0.117**
		FA	0.177	0.104	0.146	**0.0862**	0.127	0.168	0.0961	0.121	0.097	0.0869
		MD	0.0916	0.0919	0.0966	0.0889	0.0901	0.0884	0.0861	0.0817	0.086	**0.0787**
		RD	0.125	0.0996	0.12	0.0959	0.109	0.12	0.0975	0.102	0.0983	**0.0907**
		V1	37.3	61.4	36.3	36.9	35.2	36.9	34.0	35.4	34.2	**33.7**
SPIN	WM	AD	0.113	0.465	0.113	0.176	0.103	0.107	0.0877	0.0835	0.0849	**0.0761**
		FA	0.0873	0.139	0.0832	0.172	0.0813	0.0808	0.0606	0.0581	0.0588	**0.0529**
		MD	0.0544	0.249	0.0606	0.0414	0.0433	0.0437	0.0478	0.0422	0.0401	**0.0386**
		RD	0.0661	0.156	0.0687	0.0797	0.054	0.0519	0.0531	0.0479	0.0455	**0.0437**
		V1	18.7	63.2	17.8	21.3	17.0	15.1	14.7	14.3	14.6	**13.9**
	GM	AD	0.0997	0.863	0.0916	0.0933	0.0716	0.0811	0.0778	0.0754	0.0671	**0.062**
		FA	0.1	0.262	0.0862	0.0847	0.0574	**0.0513**	0.0573	0.0588	0.0564	0.0521
		MD	0.0474	0.329	0.0565	0.0473	0.0439	0.066	0.0523	0.0484	0.043	**0.0398**
		RD	0.0633	0.104	0.0687	0.0574	0.049	0.0712	0.0563	0.0533	0.0501	**0.0465**
		V1	31.0	58.0	30.5	38.7	30.3	29.1	27.9	27.8	27.7	**26.8**
AHA	WM	AD	0.155	0.73	0.11	0.118	0.115	0.145	0.0971	**0.0963**	0.106	0.1
		FA	0.0919	0.194	0.0779	0.095	0.0796	0.0889	0.0695	0.0707	0.0839	**0.0683**
		MD	0.0444	0.263	0.0404	**0.0354**	0.0356	0.0417	0.0371	0.0376	0.0401	0.0369
		RD	0.0523	0.135	0.0454	0.0458	0.0435	0.0502	0.0424	0.0432	0.0529	**0.0392**
		V1	21.9	61.2	21.1	19.9	20.4	21.3	18.4	19.4	18.1	**17.6**
	GM	AD	0.228	1.43	0.124	0.104	0.165	0.211	0.105	0.121	0.103	**0.0965**
		FA	0.133	0.303	0.105	0.08	0.106	0.128	0.0802	0.104	0.0762	**0.0748**
		MD	0.0608	0.441	0.0464	**0.0459**	0.0507	0.0559	0.049	0.0479	0.0592	0.047
		RD	0.0788	0.184	0.0692	**0.0541**	0.0658	0.0752	0.0588	0.065	0.0667	0.0556
		V1	36.0	64.3	36.3	37.8	35.1	35.6	33.7	35.0	33.4	**33.0**

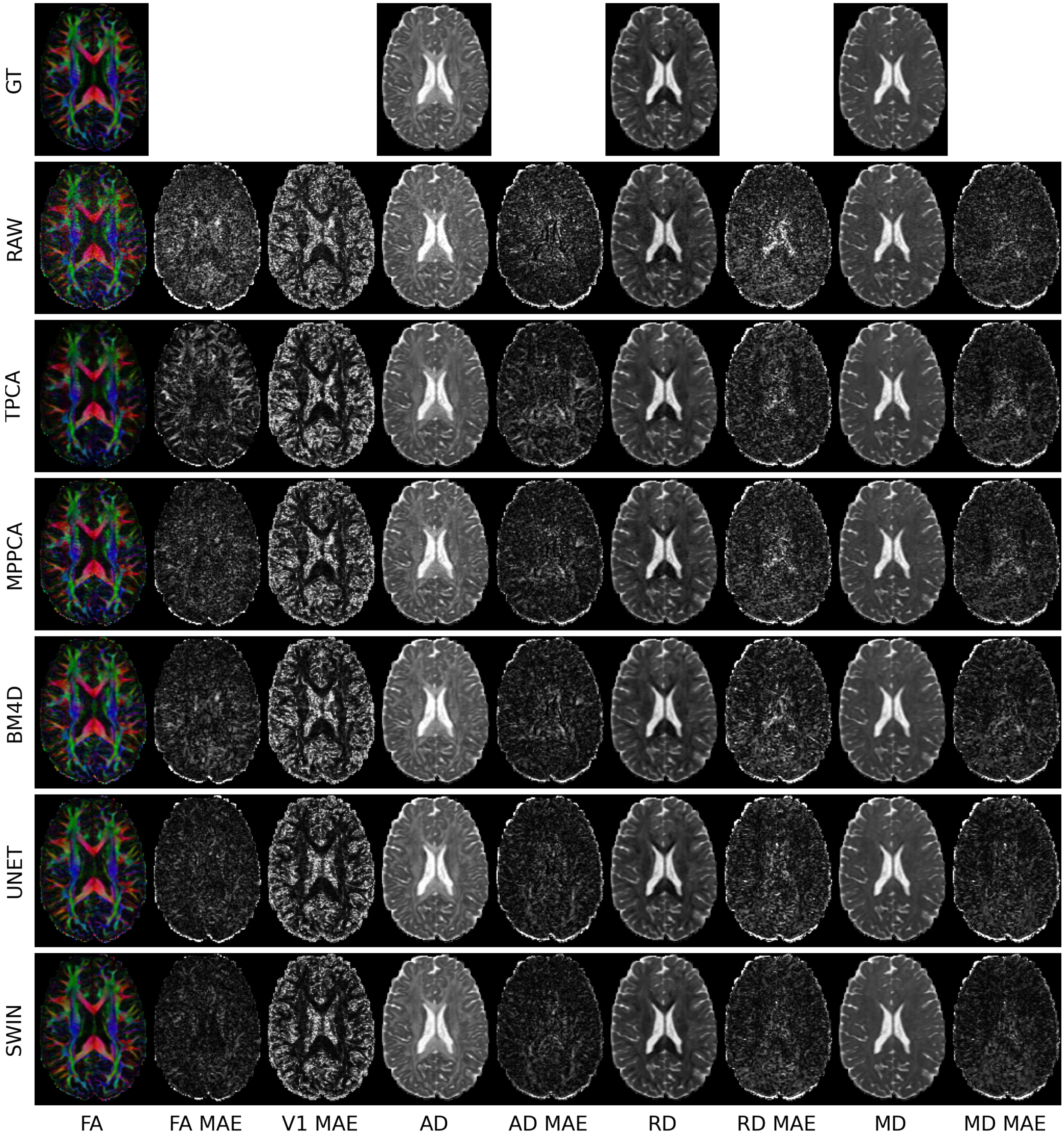

Fig. S1: Visual comparison between the ground truth (GT), no denoising (RAW), TPCA, MPPCA, BM4D, Unet, and SWIN without finetuning (SWIN) for denoising on validation data from the HCP dataset. The groundtruth is based on the full set of gradient-encoding directions at b=1000 s/mm^2 whereas the RAW, TPCA, MPPCA, BM4D, Unet, and SWIN images are derived from the six-direction subset. The mean absolute error (MAE) maps for each parameter are displayed to demonstrate the accuracy of each method. Lower MAE values signify higher accuracy and MAE maps that display anatomical structure could indicate denoising biases.

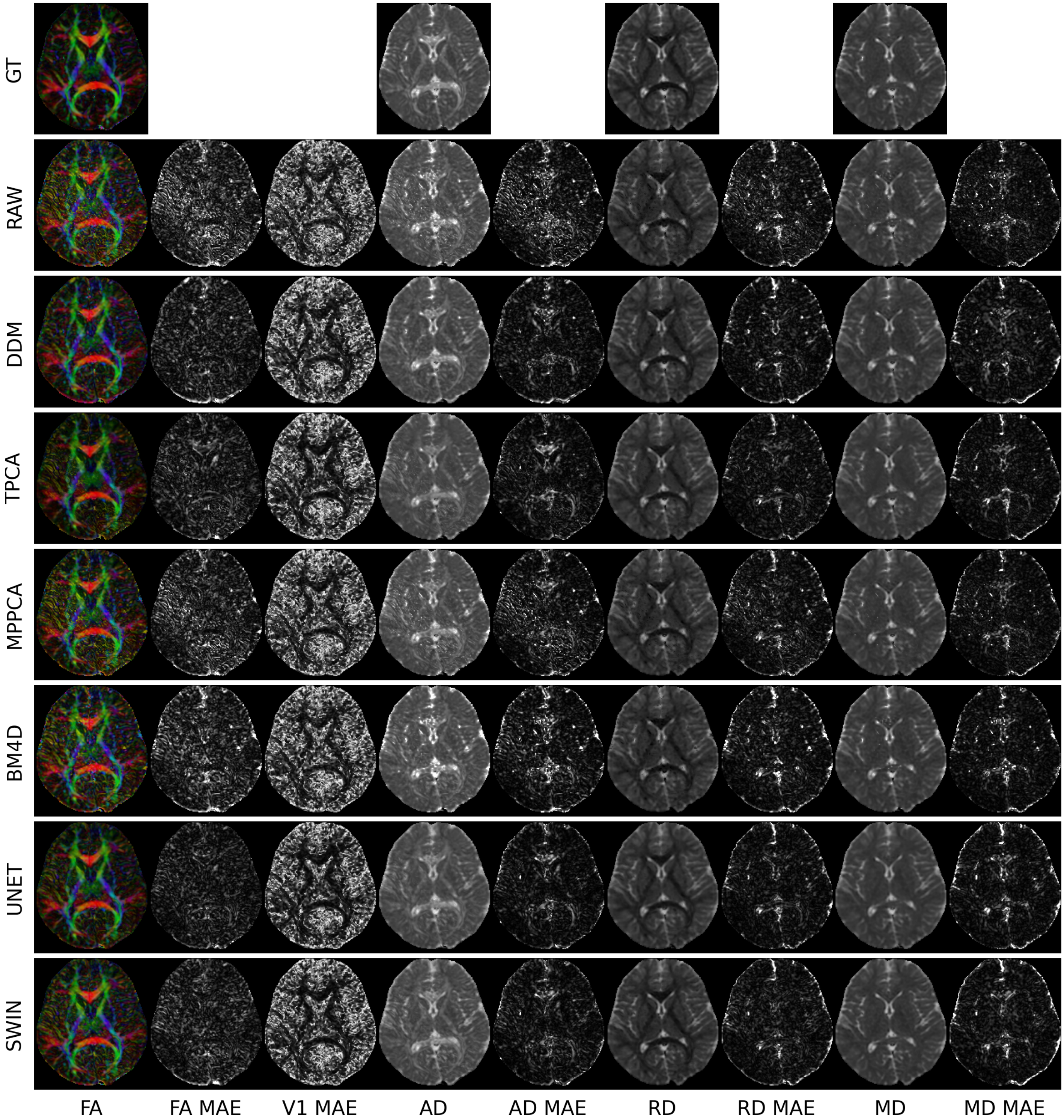

Fig. S2: Visual comparison between the ground truth (GT), no denoising (RAW), DDM^2, TPCA, BM4D, MPPCA, UNET, and SWIN without finetuning (SWIN) for denoising on validation data from the AHA dataset. The groundtruth is based on the full set of gradient-encoding directions at b=2000 s/mm^2 whereas the RAW, DDM^2, TPCA, BM4D, MPPCA, Unet, and SWIN images are derived from the six-direction subset. The mean absolute error (MAE) maps for each parameter are displayed to demonstrate the accuracy of each method. Lower MAE values signify higher accuracy and MAE maps that display anatomical structure could indicate denoising biases.

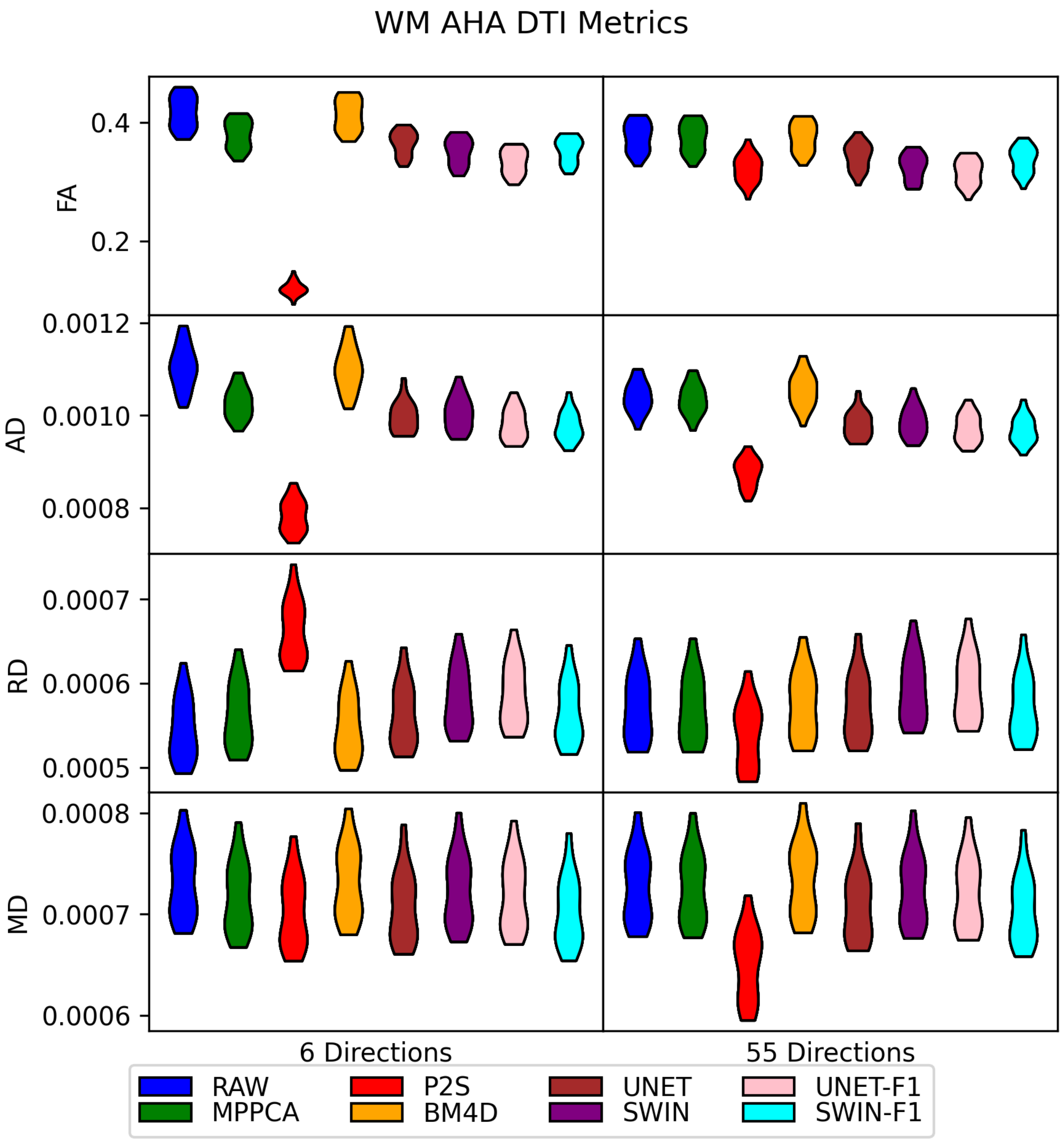

Fig. S3: DTI metrics in WM and GM from no denoising (RAW), MPPCA, BM4D, and SWIN denoising for 6 direction and 55 direction subsets from the AHA b=2000 s/mm^2 shell.

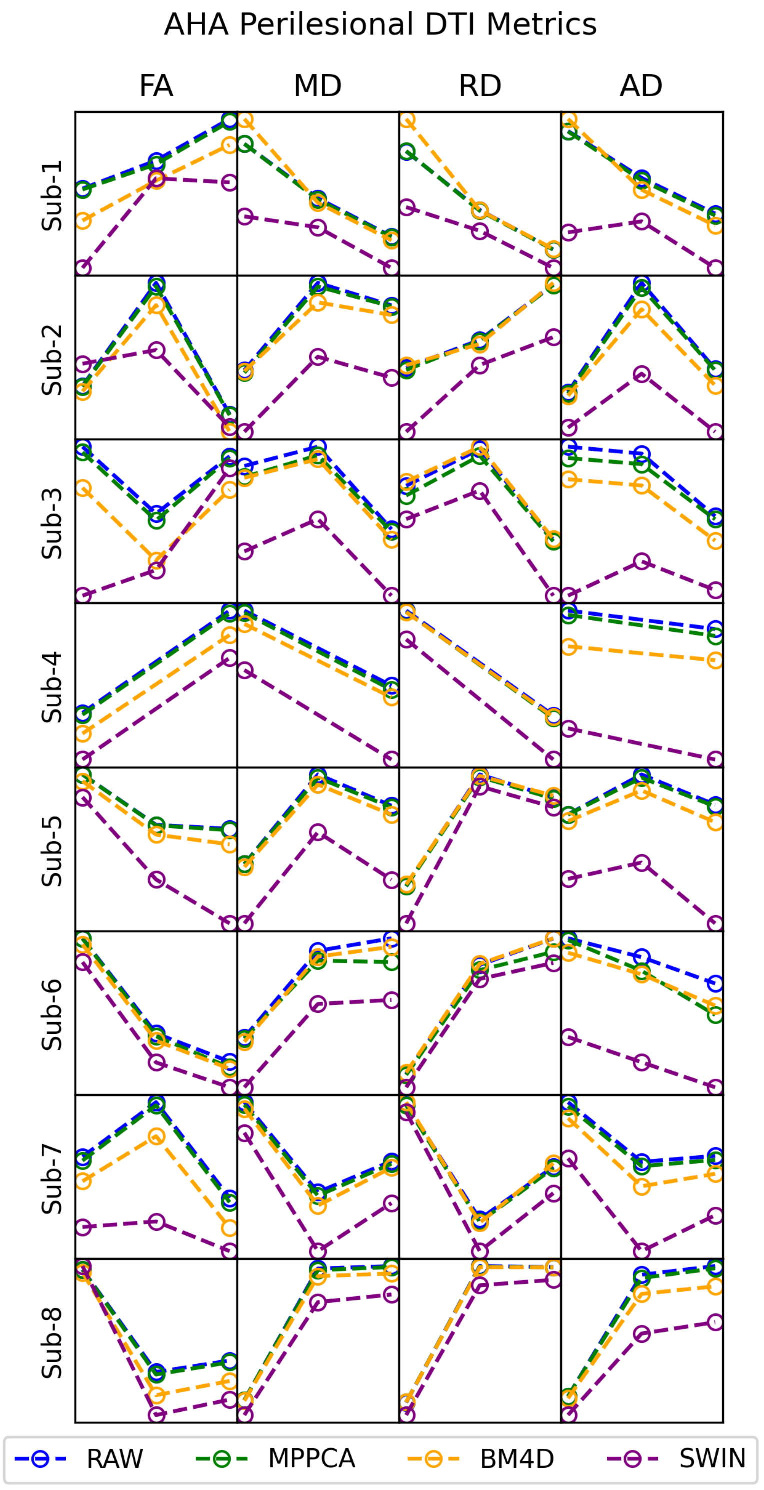

Fig. S4: Average DTI metrics (AD, FA, MD, RD) from denoising dMRI data in the perilesional space of AHA subjects across three sessions: first session data is taken one day prior to AVM resection, second session data is taken 6 months after AVM resection, and third session is taken one year after AVM resection. Subject 4 has data from only sessions one and three.

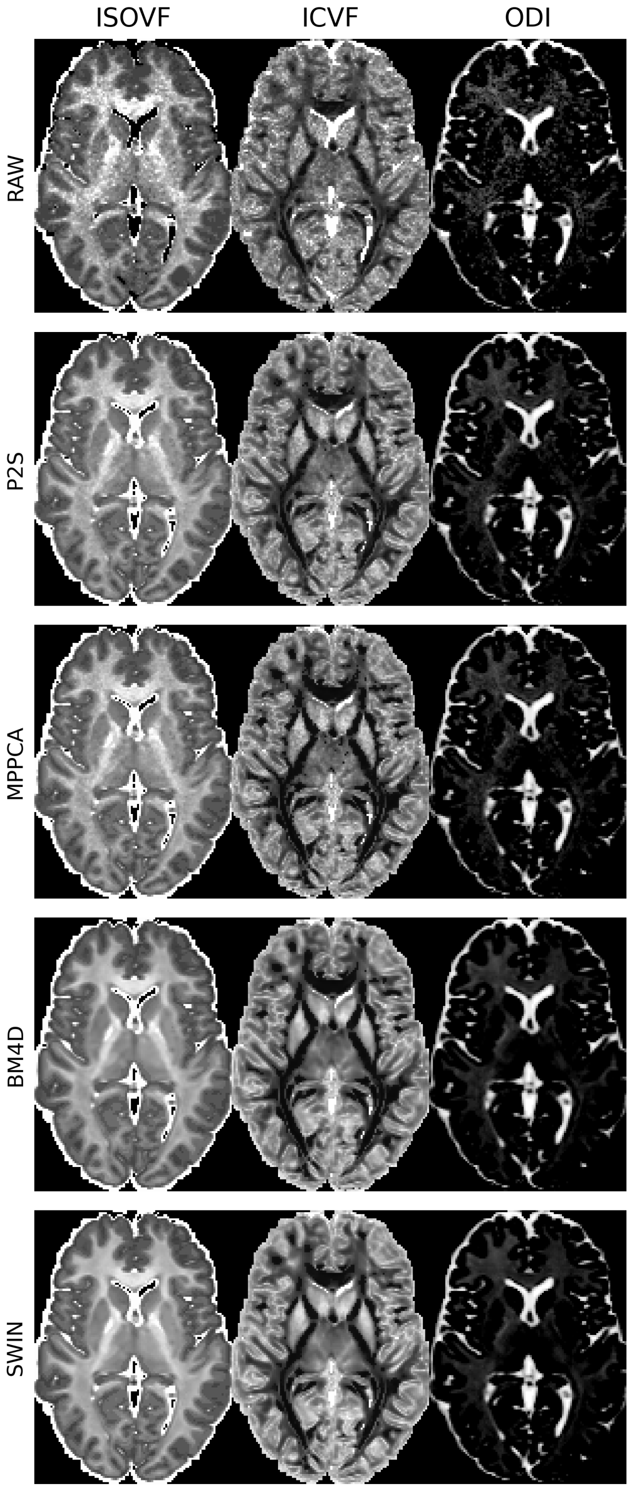

Fig. S5: Visual comparison between no denoising (RAW), BM4D, MPPCA, P2S, and SWIN for NODDI metrics on a validation subject from the HCP dataset.

For 4th and 6th order spherical harmonic fitting, the SWIN model achieves the lowest JSD in GM across all datasets (Table S7). In particular, the SWIN model outperforms all other denoising methods in the external datasets (AHA, TBI, SPIN) with the exception of 6th order estimation of B1000 WM spherical harmonics in SPIN and B2000 WM spherical harmonics in AHA, where no denoising has the lowest JSD. For the HCP dataset, the MPPCA had a lower JSD than the SWIN model for 6th order spherical harmonic estimation in higher shells. The Unet model achieves lower JSD than the SWIN model in several metrics in the HCP dataset, but performs worse in the external datasets. In some cases, applying no denoising leads to the lowest MAE. We believe this is because all denoising algorithms make assumptions to some extent and in some settings those assumptions are ill-founded.

Table S7: JSD between ground truth and estimation using 15-direction (4th order) and 28-direction (6th order) HCP, SPIN, TBI, and AHA data in white matter (WM) and gray matter (GM) via no denoising (RAW), P2S, BM4D, MPPCA, UNET, and SWIN (with and without no finetuning). Best results are **bolded**.

Site	Order	Shell	Tissue	RAW	P2S	MPPCA	BM4D	UNET	SWIN	UNET-F1	SWIN-F1
HCP	4th	B1000	WM	0.0279	0.0385	0.0264	0.0257	**0.0246**	0.0251		
			GM	0.0232	0.023	0.0208	0.0208	0.019	**0.0184**		
		B2000	WM	0.0423	0.0627	0.0396	0.0382	**0.038**	0.0383		
			GM	0.0365	0.0396	0.0317	0.0309	0.0292	**0.0286**		
		B3000	WM	0.0522	0.0742	0.0477	0.0459	0.0461	**0.0458**		
			GM	0.0486	0.047	0.0401	0.039	0.0371	**0.0364**		
	6th	B1000	WM	0.0206	0.0268	0.0203	0.0209	**0.0199**	0.0205		
			GM	0.0179	0.019	0.0173	0.0178	0.0158	**0.0157**		
		B2000	WM	0.0309	0.0432	**0.03**	0.0306	0.0307	0.0313		
			GM	0.0284	0.0307	0.0266	0.027	**0.0249**	0.0252		
		B3000	WM	0.0378	0.0526	**0.036**	0.0365	0.0375	0.0376		
			GM	0.0377	0.0383	0.0342	0.0345	**0.0322**	0.0327		
TBI	4th	B1000	WM	0.0395	0.0566	0.0396	0.0408	0.0356	**0.0354**	0.0369	0.0363
			GM	0.0361	0.0357	0.0361	0.0374	0.0311	0.0319	0.0313	**0.0305**
	6th	B1000	WM	0.0269	0.0408	0.0278	0.0284	0.0274	**0.0262**	0.0288	0.0287
			GM	0.026	0.03	0.0269	0.0277	0.0247	**0.0244**	0.0248	0.0247
SPIN	4th	B1000	WM	0.0253	0.0452	0.0256	0.0318	0.0242	0.0242	0.0255	**0.0234**
			GM	0.021	0.0323	0.0211	0.0228	0.0191	0.0191	0.019	**0.0184**
		B2500	WM	0.0481	0.0909	0.0472	0.0526	0.0461	0.0449	0.0468	**0.0436**
			GM	0.0392	0.073	0.0362	0.0377	0.0336	0.0334	0.0337	**0.0328**
	6th	B1000	WM	**0.0179**	0.0303	0.019	0.0282	0.0189	0.0187	0.0204	0.0183
			GM	0.0154	0.0243	0.0164	0.0208	0.0151	0.0149	0.0152	**0.0147**
		B2500	WM	0.0344	0.056	0.0347	0.0442	0.0356	0.0343	0.0368	**0.0338**
			GM	0.0301	0.0474	0.0293	0.0344	0.0278	**0.0273**	0.0283	0.0277
AHA	4th	B2000	WM	0.0483	0.0767	0.0482	0.0489	0.0469	**0.0457**	0.0501	0.0474
			GM	0.0429	0.0597	0.0424	0.0431	0.0388	0.0397	0.0386	**0.0382**
	6th	B2000	WM	**0.0301**	0.0537	0.0309	0.0313	0.0328	0.0305	0.0368	0.0346
			GM	0.0299	0.0523	0.0307	0.031	0.0301	**0.0295**	0.0307	0.0307

The SWIN model is able to achieve lower byte-lengths of dMRI data compressed with zlib using both high and low compression levels (Table S8).

Table S8: The sum of all dMRI data byte-lengths in Gb for the HCP test-retest diffusion dataset undergoing no denoising (RAW), Patch2Self (P2S), MPPCA, BM4D, and SWIN denoising compressed with lzib, using the DEFLATE algorithm, with compression levels of 1 (lowest) and 9 (highest). Best results are **bolded**.

Compression Level	RAW	P2S	MPPCA	BM4D	SWIN
1	64.3381	64.0960	64.3384	64.1420	**64.0651**
9	62.5757	62.2917	62.5755	62.3479	**62.2508**

Table S9: Coefficient of variation (%) for FA estimation via no denoising (RAW), P2S, BM4D, MPPCA, and SWIN for gray matter cortical regions in HCP test-retest data. Best results are **bolded**.

Cortical Regions	GT	RAW	P2S	MPPCA	BM4D	SWIN
lh-bankssts	2.172	4.164	5.232	5.394	4.023	**3.46**
lh-caudalanteriorcingulate	2.802	5.007	6.701	6.667	4.382	**4.095**
lh-caudalmiddlefrontal	3.514	4.353	5.843	5.293	3.614	**3.521**
lh-cuneus	2.675	4.051	5.03	6.597	4.95	**3.906**
lh-entorhinal	3.157	**4.179**	7.537	8.49	5.477	5.085
lh-fusiform	1.889	3.047	4.621	5.696	3.455	**3.032**
lh-inferiorparietal	1.914	3.867	4.831	4.777	3.73	**3.492**
lh-inferiortemporal	1.697	**4.773**	6.379	8.81	6.449	6.011
lh-isthmuscingulate	2.853	3.817	5.559	4.998	3.717	**3.434**
lh-lateraloccipital	1.654	5.627	6.669	6.512	5.329	**5.046**
lh-lateralorbitofrontal	2.88	**4.293**	6.806	8.264	6.126	5.416
lh-lingual	1.838	4.395	5.43	5.654	4.015	**3.81**
lh-medialorbitofrontal	4.268	**5.172**	7.835	11.19	8.096	7.244
lh-middletemporal	1.785	3.351	5.036	5.292	3.855	**3.137**
lh-parahippocampal	2.998	3.247	5.219	4.891	**3.174**	3.282
lh-paracentral	2.788	4.242	5.375	4.333	3.319	**3.004**
lh-parsopercularis	1.966	4.545	5.79	5.412	3.873	**3.503**
lh-parsorbitalis	2.785	**5.035**	8.17	8.675	6.46	5.678
lh-parstriangularis	2.673	5.211	6.794	6.327	5.119	**4.683**
lh-pericalcarine	3.738	4.472	5.2	5.83	4.284	**3.715**
lh-postcentral	2.074	4.458	5.112	3.899	3.582	**3.251**
lh-posteriorcingulate	1.928	4.368	5.797	5.541	**3.221**	3.283
lh-precentral	2.438	4.124	5.052	3.538	2.947	**2.823**
lh-precuneus	1.29	3.66	4.392	4.286	3.076	**2.436**
lh-rostralanteriorcingulate	2.155	4.06	5.689	6.188	3.87	**3.652**
lh-rostralmiddlefrontal	2.878	5.38	6.803	6.284	5.29	**4.746**
lh-superiorfrontal	2.057	4.547	5.484	4.945	3.38	**3.09**
lh-superiorparietal	3.014	3.949	4.874	4.674	3.571	**3.418**
lh-superiortemporal	1.687	**3.181**	4.776	4.482	3.31	3.297
lh-supramarginal	1.6	3.783	4.945	3.909	3.41	**3.074**
lh-frontalpole	3.495	10.45	11.46	14.11	11.6	**10.4**
lh-temporalpole	3.84	**5.668**	7.955	9.413	6.697	6.286
lh-transversetemporal	2.492	**3.941**	4.808	6.848	4.606	4.2
lh-insula	2.699	**3.06**	4.275	3.823	3.256	3.333
rh-bankssts	1.679	3.502	4.737	5.036	3.836	**3.36**
rh-caudalanteriorcingulate	3.754	3.832	5.278	5.682	3.953	**3.577**
rh-caudalmiddlefrontal	2.839	4.993	6.568	4.9	3.655	**3.624**
rh-cuneus	3.285	3.761	4.869	4.768	**3.511**	3.783
rh-entorhinal	2.655	**5.17**	6.853	10.34	7.656	7.398
rh-fusiform	1.556	**3.004**	4.721	7.11	4.116	3.803
rh-inferiorparietal	1.577	4.095	5.737	3.827	3.447	**3.249**
rh-inferiortemporal	2.19	**4.019**	5.732	9.235	5.782	5.198
rh-isthmuscingulate	3.297	3.538	5.597	5.21	3.605	**3.295**
rh-lateraloccipital	1.483	5.326	6.906	6.12	5.093	**4.467**
rh-lateralorbitofrontal	3.025	**3.649**	6.27	7.66	4.896	4.461
rh-lingual	2.591	3.648	5.079	5.146	**3.422**	3.49
rh-medialorbitofrontal	3.865	**5.22**	6.977	9.224	7.26	6.678
rh-middletemporal	2.347	4.295	5.019	6.99	4.56	**4.009**
rh-parahippocampal	2.034	**3.577**	5.011	6.949	3.945	4.267
rh-paracentral	5.078	**3.526**	4.215	5.596	3.67	4.639
rh-parsopercularis	2.042	3.875	5.308	4.527	2.995	**2.881**
rh-parsorbitalis	3.394	**6.082**	7.149	10.66	7.513	6.845
rh-parstriangularis	3.418	4.89	5.84	6.77	4.735	**4.57**
rh-pericalcarine	2.601	3.61	4.901	4.31	3.816	**3.304**
rh-postcentral	2.064	4.4	5.181	3.945	3.16	**2.99**
rh-posteriorcingulate	3.623	3.635	5.461	5.343	**3.468**	3.577
rh-precentral	2.099	3.926	4.932	3.335	**2.391**	2.568

rh-precuneus	3.332	3.356	4.715	4.021	2.689	**2.654**
rh-rostralanteriorcingulate	2.957	3.886	5.155	5.762	3.591	**3.376**
rh-rostralmiddlefrontal	3.14	5.465	6.431	6.758	4.821	**4.266**
rh-superiorfrontal	2.647	4.873	5.732	5.123	3.198	**3.076**
rh-superiorparietal	1.542	4.339	5.296	4.48	3.519	**3.242**
rh-superiortemporal	1.798	**2.978**	4.381	5.179	3.259	3.086
rh-supramarginal	1.828	3.413	4.906	3.71	2.767	**2.47**
rh-frontalpole	3.748	**9.115**	11.02	14.46	11.46	10.14
rh-temporalpole	3.801	**6.287**	7.234	12.14	8.362	7.879
rh-transversetemporal	3.085	**3.805**	4.704	7.426	4.481	4.681
rh-insula	1.654	2.262	3.813	3.394	2.082	**1.879**

Table S10: Coefficient of variation (%) for MD estimation via no denoising (RAW), P2S, BM4D, MPPCA, and SWIN for gray matter cortical regions in HCP test-retest data. Best results are **bolded**.

Cortical Regions	GT	RAW	P2S	MPPCA	BM4D	SWIN
lh-bankssts	1.072	2.048	2.02	2.054	2.022	**1.957**
lh-caudalanteriorcingulate	1.677	2.419	2.409	2.427	2.361	**2.331**
lh-caudalmiddlefrontal	1.333	2.277	2.308	2.274	2.243	**2.122**
lh-cuneus	1.498	2.096	**2.08**	2.092	2.081	2.108
lh-entorhinal	2.644	**4.279**	4.369	4.381	4.351	4.296
lh-fusiform	0.9479	1.732	1.759	1.755	1.776	**1.67**
lh-inferiorparietal	1.415	2.292	2.27	2.294	2.302	**2.195**
lh-inferiortemporal	1.121	2.799	2.835	2.828	2.821	**2.639**
lh-isthmuscingulate	1.486	2.491	2.483	2.458	2.477	**2.454**
lh-lateraloccipital	1.475	2.522	2.598	2.548	2.565	**2.507**
lh-lateralorbitofrontal	1.771	**3.255**	3.404	3.427	3.426	3.306
lh-lingual	1.512	1.895	1.888	1.876	1.919	**1.828**
lh-medialorbitofrontal	2.745	4.605	4.695	4.801	4.774	**4.31**
lh-middletemporal	1.312	2.328	2.357	2.341	2.326	**2.292**
lh-parahippocampal	1.178	2.675	**2.625**	2.657	2.68	2.684
lh-paracentral	1.781	2.297	2.294	2.285	2.244	**2.128**
lh-parsopercularis	1.403	1.862	1.882	1.852	1.84	**1.788**
lh-parsorbitalis	1.666	3.204	3.168	3.224	3.25	**2.982**
lh-parstriangularis	1.702	1.948	2.011	1.954	1.962	**1.941**
lh-pericalcarine	1.46	2.141	2.148	2.133	2.099	**2.07**
lh-postcentral	1.534	2.141	2.131	2.139	2.092	**2.055**
lh-posteriorcingulate	1.389	2.045	2.094	2.065	2.03	**1.922**
lh-precentral	1.402	2.023	2.056	2.023	2.015	**1.923**
lh-precuneus	1.16	1.828	1.789	1.815	1.788	**1.781**
lh-rostralanteriorcingulate	1.736	2.739	2.725	2.751	2.734	**2.656**
lh-rostralmiddlefrontal	1.479	2.132	2.098	2.144	2.122	**2.062**
lh-superiorfrontal	1.293	1.713	1.741	1.717	**1.678**	1.685
lh-superiorparietal	1.531	2.322	2.3	2.309	2.297	**2.219**
lh-superiortemporal	1.084	2.021	2.043	2.008	2.017	**1.972**
lh-supramarginal	1.197	1.878	1.87	1.872	1.86	**1.836**
lh-frontalpole	4.041	7.534	7.535	7.627	7.503	**7.034**
lh-temporalpole	3.18	**3.808**	3.881	3.869	3.933	3.849
lh-transversetemporal	1.608	2.614	2.578	2.585	2.513	**2.489**
lh-insula	1.035	1.917	1.913	1.921	1.912	**1.909**
rh-bankssts	1.127	2.185	2.184	2.18	2.177	**2.13**
rh-caudalanteriorcingulate	1.12	1.419	1.455	1.412	1.411	**1.403**
rh-caudalmiddlefrontal	1.558	2.149	2.177	2.135	2.077	**1.943**
rh-cuneus	1.478	2.446	2.443	2.428	**2.404**	2.418
rh-entorhinal	1.971	3.506	3.58	3.57	3.544	**3.465**
rh-fusiform	1.474	2.265	2.261	2.231	2.281	**2.13**
rh-inferiorparietal	1.337	2.23	2.207	2.208	2.178	**2.108**
rh-inferiortemporal	1.989	2.687	2.803	2.757	2.78	**2.569**

rh-isthmuscingulate	1.073	2.137	2.092	2.135	2.13	**2.086**
rh-lateraloccipital	1.818	2.524	2.461	2.498	2.493	**2.365**
rh-lateralorbitofrontal	2.297	**3.217**	3.227	3.281	3.332	3.25
rh-lingual	1.389	2.401	2.363	2.399	2.384	**2.328**
rh-medialorbitofrontal	2.521	4.382	4.53	4.591	4.662	**4.196**
rh-middletemporal	1.639	2.55	2.568	2.551	2.552	**2.493**
rh-parahippocampal	0.9239	2.308	2.325	2.302	2.294	**2.169**
rh-paracentral	2.01	2.207	2.178	2.191	2.212	**2.132**
rh-parsopercularis	1.481	1.801	**1.755**	1.796	1.781	1.873
rh-parsorbitalis	2.493	3.234	**3.101**	3.217	3.269	3.108
rh-parstriangularis	2.04	2.874	**2.835**	2.872	2.841	2.893
rh-pericalcarine	1.463	2.114	2.127	2.123	**2.084**	2.127
rh-postcentral	1.334	1.617	1.61	1.616	1.572	**1.477**
rh-posteriorcingulate	1.159	1.675	1.69	1.676	1.675	**1.578**
rh-precentral	1.38	1.847	1.853	1.842	1.79	**1.666**
rh-precuneus	1.156	1.737	1.71	1.729	1.715	**1.701**
rh-rostralanteriorcingulate	1.4	1.961	1.951	1.961	1.942	**1.875**
rh-rostralmiddlefrontal	1.934	2.609	**2.554**	2.605	2.574	2.594
rh-superiorfrontal	1.334	1.844	1.88	1.829	1.8	**1.656**
rh-superiorparietal	1.422	2.38	2.373	2.378	2.336	**2.256**
rh-superiortemporal	1.363	1.717	1.741	1.712	**1.679**	1.732
rh-supramarginal	1.203	1.921	1.899	1.913	1.87	**1.836**
rh-frontalpole	3.252	6.283	6.368	6.38	6.38	**5.716**
rh-temporalpole	2.565	**2.85**	3.062	3.146	3.18	3.192
rh-transversetemporal	1.749	2.591	2.564	2.573	**2.503**	2.525
rh-insula	1.135	1.689	1.686	1.674	1.692	**1.665**

Table S11: Coefficient of variation (%) for RD estimation via no denoising (RAW), P2S, BM4D, MPPCA, and SWIN for gray matter cortical regions in HCP test-retest data. Best results are **bolded**.

Cortical Regions	GT	RAW	P2S	MPPCA	BM4D	SWIN
lh-bankssts	1.098	2.347	2.138	2.268	2.247	**2.023**
lh-caudalanteriorcingulate	1.71	2.912	2.624	2.689	2.603	**2.51**
lh-caudalmiddlefrontal	1.422	2.41	2.322	2.343	2.323	**2.229**
lh-cuneus	1.624	2.36	**2.105**	2.356	2.334	2.335
lh-entorhinal	2.76	4.89	4.634	4.642	4.687	**4.591**
lh-fusiform	0.9626	1.898	1.831	1.964	1.931	**1.782**
lh-inferiorparietal	1.464	2.375	**2.314**	2.418	2.393	2.331
lh-inferiortemporal	1.109	3.07	2.95	3.069	3.079	**2.878**
lh-isthmuscingulate	1.699	2.828	**2.572**	2.878	2.845	2.721
lh-lateraloccipital	1.507	2.808	2.684	**2.679**	2.703	2.688
lh-lateralorbitofrontal	1.774	3.952	**3.587**	3.809	3.832	3.717
lh-lingual	1.577	2.159	1.983	2.064	2.048	**1.946**
lh-medialorbitofrontal	2.774	5.154	4.945	4.889	5.041	**4.539**
lh-middletemporal	1.361	2.46	2.43	2.439	2.452	**2.425**
lh-parahippocampal	1.411	2.931	**2.744**	2.912	2.888	2.89
lh-paracentral	1.907	2.381	2.288	2.362	2.3	**2.197**
lh-parsopercularis	1.473	2.019	**1.891**	1.955	1.921	1.906
lh-parsorbitalis	1.693	3.678	3.341	3.7	3.678	**3.286**
lh-parstriangularis	1.775	2.371	**2.014**	2.334	2.326	2.237
lh-pericalcarine	1.612	2.331	2.186	2.286	2.184	**2.163**
lh-postcentral	1.592	2.229	2.138	2.172	2.15	**2.1**
lh-posteriorcingulate	1.469	2.47	2.241	2.201	2.163	**2.066**
lh-precentral	1.528	2.122	2.066	2.124	2.117	**2.039**
lh-precuneus	1.229	1.864	**1.799**	1.865	1.827	1.855
lh-rostralanteriorcingulate	1.735	3.135	**2.814**	3.037	2.966	2.835
lh-rostralmiddlefrontal	1.46	2.415	**2.21**	2.319	2.333	2.218
lh-superiorfrontal	1.241	1.931	**1.773**	1.842	1.812	1.789

lh-superiorparietal	1.623	2.34	**2.3**	2.409	2.358	2.347
lh-superiortemporal	1.128	2.063	2.082	2.086	2.085	**2.042**
lh-supramarginal	1.254	1.951	**1.894**	1.959	1.934	1.908
lh-frontalpole	3.976	8.321	7.799	7.921	7.903	**7.384**
lh-temporalpole	3.229	4.178	**4.07**	4.151	4.199	4.08
lh-transversetemporal	1.699	2.691	2.63	2.605	2.577	**2.509**
lh-insula	1.19	2.281	**1.969**	2.162	2.143	2.11
rh-bankssts	1.176	2.238	2.225	2.203	2.225	**2.195**
rh-caudalanteriorcingulate	1.243	1.533	**1.433**	1.513	1.531	1.518
rh-caudalmiddlefrontal	1.618	2.343	2.286	2.187	2.171	**2.056**
rh-cuneus	1.672	2.562	**2.447**	2.615	2.574	2.591
rh-entorhinal	2.016	4.323	**3.679**	4.179	4.182	4.056
rh-fusiform	1.529	2.489	2.395	2.554	2.498	**2.358**
rh-inferiorparietal	1.396	2.368	2.267	2.375	2.335	**2.233**
rh-inferiortemporal	2.091	3.077	2.893	3.097	3.088	**2.86**
rh-isthmuscingulate	1.382	2.464	**2.225**	2.406	2.395	2.343
rh-lateraloccipital	1.898	2.731	2.592	2.689	2.703	**2.497**
rh-lateralorbitofrontal	2.328	3.494	3.44	**3.336**	3.455	3.438
rh-lingual	1.502	2.675	**2.486**	2.561	2.532	2.53
rh-medialorbitofrontal	2.563	5.088	4.617	4.8	5.024	**4.521**
rh-middletemporal	1.734	2.838	**2.666**	2.865	2.824	2.69
rh-parahippocampal	1.021	2.563	2.514	2.459	2.464	**2.378**
rh-paracentral	2.357	2.267	**2.177**	2.442	2.391	2.384
rh-parsopercularis	1.496	1.953	**1.763**	1.925	1.882	1.948
rh-parsorbitalis	2.54	3.588	**3.174**	3.536	3.51	3.391
rh-parstriangularis	2.051	3.026	2.926	**2.878**	2.912	2.919
rh-pericalcarine	1.567	2.331	**2.2**	2.268	2.266	2.278
rh-postcentral	1.322	1.741	1.682	1.663	1.61	**1.492**
rh-posteriorcingulate	1.33	1.885	1.836	1.715	1.732	**1.681**
rh-precentral	1.43	1.912	1.898	1.863	1.8	**1.692**
rh-precuneus	1.337	1.756	**1.705**	1.788	1.798	1.807
rh-rostralanteriorcingulate	1.391	2.282	**2.007**	2.188	2.159	2.056
rh-rostralmiddlefrontal	1.846	2.832	**2.619**	2.715	2.7	2.651
rh-superiorfrontal	1.31	1.802	1.87	1.752	1.735	**1.671**
rh-superiorparietal	1.504	2.538	2.417	2.488	2.458	**2.385**
rh-superiortemporal	1.392	1.881	**1.818**	1.937	1.841	1.821
rh-supramarginal	1.241	2.067	1.977	2.061	1.98	**1.925**
rh-frontalpole	3.222	7.201	6.628	6.965	7.015	**6.211**
rh-temporalpole	2.607	3.797	**3.201**	4.099	3.99	3.87
rh-transversetemporal	1.846	2.774	**2.636**	2.746	2.664	2.675
rh-insula	1.198	1.82	1.758	1.834	1.826	**1.751**

Table S12: Coefficient of variation (%) for AD estimation via no denoising (RAW), P2S, BM4D, MPPCA, and SWIN for gray matter cortical regions in HCP test-retest data. Best results are **bolded**.

Cortical Regions	GT	RAW	P2S	MPPCA	BM4D	SWIN
lh-bankssts	1.121	1.997	1.934	2.172	1.976	**1.887**
lh-caudalanteriorcingulate	1.653	**1.933**	2.078	2.113	2.063	2.12
lh-caudalmiddlefrontal	1.31	2.219	2.304	2.233	2.15	**2.003**
lh-cuneus	1.311	1.914	2.043	1.831	1.834	**1.8**
lh-entorhinal	2.502	**3.704**	3.95	4.159	3.93	3.935
lh-fusiform	0.9601	1.765	1.694	1.812	1.766	**1.551**
lh-inferiorparietal	1.37	2.196	2.191	2.1	2.164	**1.994**
lh-inferiortemporal	1.196	2.824	**2.678**	3.285	2.936	2.686
lh-isthmuscingulate	1.241	2.249	2.38	**1.989**	2.067	2.104
lh-lateraloccipital	1.449	2.431	2.506	2.573	2.563	**2.366**
lh-lateralorbitofrontal	1.785	**2.67**	3.137	3.296	3.023	2.773
lh-lingual	1.423	1.868	1.826	1.78	1.81	**1.729**

lh-medialorbitofrontal	2.699	**4.136**	4.41	4.903	4.557	4.186
lh-middletemporal	1.286	2.208	2.248	2.253	2.179	**2.113**
lh-parahippocampal	0.965	2.612	2.526	2.525	2.505	**2.469**
lh-paracentral	1.597	2.459	2.356	2.235	2.262	**2.043**
lh-parsopercularis	1.348	1.954	1.888	1.877	1.831	**1.736**
lh-parsorbitalis	1.724	3.114	2.949	3.143	3.131	**2.864**
lh-parstriangularis	1.658	2.025	2.017	2.037	2.008	**1.879**
lh-pericalcarine	1.305	2.199	2.149	2.119	2.077	**1.987**
lh-postcentral	1.491	2.12	2.138	2.118	2.042	**2.01**
lh-posteriorcingulate	1.272	**1.652**	1.882	2.098	1.928	1.755
lh-precentral	1.265	1.913	2.038	1.887	1.874	**1.763**
lh-precuneus	1.092	1.957	1.826	1.774	1.776	**1.7**
lh-rostralanteriorcingulate	1.779	2.468	2.617	2.431	2.428	**2.404**
lh-rostralmiddlefrontal	1.715	2.239	2.138	2.355	2.229	**2.006**
lh-superiorfrontal	1.41	1.834	1.874	1.87	1.774	**1.611**
lh-superiorparietal	1.417	2.306	2.303	2.151	2.192	**2.012**
lh-superiortemporal	1.045	2.066	2.042	1.952	1.959	**1.874**
lh-supramarginal	1.183	1.921	1.85	1.823	1.834	**1.789**
lh-frontalpole	4.156	6.732	7.093	7.376	6.966	**6.481**
lh-temporalpole	3.138	4.148	**3.804**	4.205	4.018	3.921
lh-transversetemporal	1.496	2.677	**2.524**	2.762	2.621	2.55
lh-insula	0.9875	1.8	1.927	1.749	1.749	**1.692**
rh-bankssts	1.085	2.234	2.165	2.265	2.179	**2.061**
rh-caudalanteriorcingulate	1.21	1.582	1.52	1.76	1.589	**1.515**
rh-caudalmiddlefrontal	1.541	2.19	2.057	2.242	2.072	**1.922**
rh-cuneus	1.227	2.356	2.464	2.223	2.196	**2.162**
rh-entorhinal	1.947	**2.926**	3.426	3.349	3.156	3.04
rh-fusiform	1.407	2.201	2.111	2.284	2.184	**1.972**
rh-inferiorparietal	1.293	2.344	2.145	2.263	2.19	**2.058**
rh-inferiortemporal	1.838	2.491	2.669	2.792	2.652	**2.339**
rh-isthmuscingulate	0.9587	1.833	1.991	1.979	1.837	**1.76**
rh-lateraloccipital	1.694	2.729	**2.328**	2.734	2.619	2.38
rh-lateralorbitofrontal	2.252	3.034	**2.986**	3.5	3.303	3.132
rh-lingual	1.265	2.227	2.281	2.355	2.253	**2.089**
rh-medialorbitofrontal	2.469	**3.872**	4.474	4.794	4.419	3.886
rh-middletemporal	1.519	2.523	2.489	2.499	2.472	**2.323**
rh-parahippocampal	0.8749	2.146	2.098	2.283	2.104	**1.94**
rh-paracentral	1.513	2.445	2.243	1.936	2.107	**1.832**
rh-parsopercularis	1.505	1.948	1.864	1.95	1.859	**1.816**
rh-parsorbitalis	2.468	3.47	**3.094**	3.688	3.603	3.237
rh-parstriangularis	2.125	2.968	**2.772**	3.083	2.901	2.883
rh-pericalcarine	1.386	2.054	2.073	2.07	1.972	**1.935**
rh-postcentral	1.375	1.759	1.558	1.698	1.615	**1.528**
rh-posteriorcingulate	1.045	1.567	1.629	1.872	1.683	**1.532**
rh-precentral	1.373	1.985	1.898	1.867	1.84	**1.676**
rh-precuneus	1.041	1.76	1.718	1.739	1.652	**1.57**
rh-rostralanteriorcingulate	1.583	1.938	1.954	1.921	1.81	**1.659**
rh-rostralmiddlefrontal	2.109	2.76	**2.501**	2.742	2.626	2.598
rh-superiorfrontal	1.444	2.171	2.037	2.08	1.98	**1.72**
rh-superiorparietal	1.338	2.217	2.292	2.225	2.157	**2.059**
rh-superiortemporal	1.361	1.897	**1.72**	1.868	1.76	1.731
rh-supramarginal	1.216	1.91	1.856	1.968	1.861	**1.785**
rh-frontalpole	3.328	5.482	5.925	6.206	5.899	**5.334**
rh-temporalpole	2.529	2.799	2.869	3.077	2.812	**2.703**
rh-transversetemporal	1.616	2.638	**2.503**	2.871	2.512	2.525
rh-insula	1.071	1.635	1.632	1.554	1.565	**1.546**

Table S13: Coefficient of variation (%) for FA estimation via no denoising (RAW), P2S, BM4D, MPPCA, and SWIN for white matter regions in HCP test-retest data. Best results are **bolded**.

White Matter Regions	GT	RAW	P2S	MPPCA	BM4D	SWIN
MCP	0.8846	**1.758**	5.121	2.407	2.195	2.154
PCT	1.274	**2.487**	5.15	3.657	3.459	3.59
GCC	0.5437	**1.189**	4.451	1.507	1.393	1.466
BCC	0.6061	**1.857**	6.761	1.903	1.918	1.964
SCC	0.549	**1.441**	7.807	1.638	1.671	1.769
FX	1.508	**2.783**	8.903	3.637	3.267	3.157
CST-R	1.583	**2.872**	8.514	3.982	3.986	4.075
CST-L	1.221	**2.093**	7.521	2.796	2.616	2.536
ML-R	1.015	**2.223**	8.401	2.695	2.487	2.613
ML-L	1.089	**2.304**	8.228	2.83	2.805	2.586
ICP-R	1.18	**2.14**	9.024	2.88	2.645	2.612
ICP-L	1.206	**2.505**	8.379	2.804	2.832	2.668
SCP-R	1.268	**2.496**	9.602	3.081	3.021	3.161
SCP-L	1.28	**2.665**	8.458	2.993	2.926	3.114
CP-R	0.7031	**1.729**	9.776	2.145	2.065	2.025
CP-L	0.6827	**2.354**	8.168	2.672	2.604	2.705
ALIC-R	1.435	**2.825**	7.687	3.627	3.271	3.359
ALIC-L	1.125	**3.037**	7.275	3.428	3.364	3.325
PLIC-R	0.9096	**1.654**	6.827	1.861	1.861	1.851
PLIC-L	0.9	**2.276**	7.927	2.605	2.468	2.581
RLIC-R	1.208	**2.01**	6.992	2.481	2.332	2.318
RLIC-L	1.088	**2.651**	8.37	3.128	3.006	3.36
ACR-R	1.079	**1.425**	5.824	2.207	1.925	1.842
ACR-L	0.7943	2.11	5.782	1.887	1.852	**1.694**
SCR-R	0.9877	**1.884**	5.467	2.328	2.2	2.187
SCR-L	1.548	5.075	7.557	5.052	4.995	**4.893**
PCR-R	0.7091	2.143	5.307	2.168	**2.097**	2.313
PCR-L	1.062	3.229	7.392	**2.999**	3.141	3.123
PTR-R	0.8196	**1.954**	8.01	2.189	2.173	2.152
PTR-L	0.9864	2.816	7.782	2.882	2.913	**2.763**
SS-R	1.096	**1.824**	8.008	2.107	1.939	2.031
SS-L	1.256	**3.011**	8.307	3.605	3.301	3.428
EC-R	1.087	**1.725**	6.079	2.411	2.146	2.204
EC-L	0.7778	1.915	5.476	1.793	**1.656**	1.724
CGC-R	1.606	**2.1**	5.282	2.87	2.569	2.509
CGC-L	1.427	3.36	7.266	3.516	3.424	**3.223**
CGH-R	1.542	**3.523**	8.564	5.393	4.313	4.564
CGH-L	2.303	**3.735**	8.204	5.637	4.42	4.656
FXST-R	1.418	**2.069**	8.61	2.857	2.727	2.781
FXST-L	1.492	**2.769**	6.629	3.342	3.198	3.359
SLF-R	0.8572	**2.1**	4.789	2.467	2.329	2.569
SLF-L	0.9807	2.952	6.985	2.973	2.948	**2.845**
SFO-R	1.743	**3.602**	9.083	5.006	4.908	4.427
SFO-L	2.151	4.429	8.205	4.484	4.463	**4.095**
UNC-R	2.527	**3.879**	9.825	5.056	4.494	4.649
UNC-L	1.687	**3.675**	8.63	4.451	3.726	3.834
TPT-R	1.335	3.155	7.296	3.2	3.121	**3.115**
TPT-L	1.465	5.129	9.922	4.702	4.621	**4.283**

Table S14: Coefficient of variation (%) for MD estimation via no denoising (RAW), P2S, BM4D, MPPCA, and SWIN for white matter regions in HCP test-retest data. Best results are **bolded**.

White Matter Regions	GT	RAW	P2S	MPPCA	BM4D	SWIN
MCP	1.115	2.468	2.793	2.456	2.493	**2.333**

PCT	1.305	2.788	3.054	2.645	2.682	**2.468**
GCC	2.778	**2.009**	2.93	2.213	2.202	2.177
BCC	1.72	**2.435**	3.093	2.617	2.622	2.457
SCC	1.683	**2.268**	3.598	2.533	2.527	2.573
FX	3.043	3.625	3.94	3.715	3.565	**3.491**
CST-R	1.183	2.555	3.085	2.529	2.527	**2.473**
CST-L	1.324	2.435	3.06	2.45	2.475	**2.317**
ML-R	1.192	2.478	2.75	**2.378**	2.397	2.4
ML-L	1.2	2.95	3.223	2.995	2.92	**2.773**
ICP-R	1.136	2.932	3.292	2.869	2.886	**2.717**
ICP-L	1.129	2.989	3.172	2.989	3.021	**2.825**
SCP-R	1.239	2.35	3.406	2.341	**2.28**	2.322
SCP-L	1.314	3.216	4.072	3.196	**3.157**	3.165
CP-R	1.563	**2.487**	3.991	2.693	2.703	2.707
CP-L	2.101	**3.488**	4.297	3.542	3.609	3.758
ALIC-R	1.833	**2.394**	3.271	2.576	2.464	2.411
ALIC-L	3.329	**4.885**	5.901	5.357	5.158	5.135
PLIC-R	0.9723	1.688	2.429	1.745	1.745	**1.672**
PLIC-L	2.127	**3.626**	4.358	3.706	3.728	3.753
RLIC-R	1.55	2.747	3.091	2.72	2.76	**2.69**
RLIC-L	2.075	2.738	2.711	2.824	2.803	**2.616**
ACR-R	1.584	1.891	2.167	1.889	1.861	**1.795**
ACR-L	2.51	**2.562**	2.812	2.632	2.583	2.57
SCR-R	1.396	2.543	2.902	**2.464**	2.545	2.514
SCR-L	2.799	4.603	4.659	4.642	4.646	**4.464**
PCR-R	0.9936	2.098	2.158	2.065	2.054	**2.035**
PCR-L	2.202	3.795	4.016	3.864	3.752	**3.486**
PTR-R	1.454	2.691	3.608	2.676	2.678	**2.629**
PTR-L	2.067	3.087	3.658	3.151	3.13	**2.928**
SS-R	2.316	3.026	3.678	3.063	3.03	**2.867**
SS-L	2.248	**2.598**	2.715	2.662	2.663	2.759
EC-R	1.298	2.444	2.821	2.462	2.434	**2.288**
EC-L	1.598	1.961	2.075	1.988	1.949	**1.906**
CGC-R	1.382	1.879	2.291	1.85	1.836	**1.703**
CGC-L	3.309	4.649	5.186	4.923	4.796	**4.582**
CGH-R	1.198	2.548	3.019	2.545	2.414	**2.359**
CGH-L	1.625	2.832	2.867	2.778	2.831	**2.772**
FXST-R	1.427	2.418	3.031	2.475	**2.313**	2.376
FXST-L	1.934	**2.497**	2.94	2.651	2.508	2.656
SLF-R	1.305	2.274	2.493	2.282	2.287	**2.27**
SLF-L	2.445	4.384	4.611	4.495	4.441	**4.152**
SFO-R	2.321	3.569	4.03	**3.282**	3.345	3.34
SFO-L	4.599	6.482	6.575	6.087	6.358	**5.989**
UNC-R	1.902	3.472	3.306	3.35	3.432	**3.134**
UNC-L	1.434	2.748	3.065	2.785	2.643	**2.328**
TPT-R	1.822	3.362	**3.212**	3.335	3.281	3.523
TPT-L	2.647	5.352	5.179	5.17	4.859	**4.607**

Table S15: Coefficient of variation (%) for RD estimation via no denoising (RAW), P2S, BM4D, MPPCA, and SWIN for white matter regions in HCP test-retest data. Best results are **bolded**.

White Matter Regions	GT	RAW	P2S	MPPCA	BM4D	SWIN
MCP	1.777	3.89	**3.39**	3.866	3.878	3.608
PCT	1.843	4.329	**3.462**	4.174	4.22	3.843
GCC	2.688	4.209	**3.269**	4.431	4.415	4.289
BCC	3.027	6.325	**4.038**	6.256	6.47	5.827
SCC	3.396	6.538	**4.377**	7.04	7.265	7.21
FX	5.346	6.488	**4.07**	6.619	6.684	6.392

CST-R	2.075	4.493	**3.807**	4.565	4.457	4.307
CST-L	2.263	3.823	3.751	3.783	3.729	**3.386**
ML-R	1.871	4.188	**3.476**	4.223	4.108	4.002
ML-L	2.231	4.896	**3.956**	4.768	4.756	4.303
ICP-R	1.745	4.517	**3.773**	4.687	4.626	4.271
ICP-L	1.684	4.9	**3.738**	4.789	4.76	4.306
SCP-R	2.758	4.367	4.609	4.411	4.233	**4.167**
SCP-L	3.269	5.961	**4.919**	5.88	5.788	5.788
CP-R	2.61	6.292	**5.319**	6.69	6.774	6.444
CP-L	3.464	7.232	**5.223**	7.151	7.39	7.548
ALIC-R	2.994	5.366	**4.228**	5.251	5.155	5.0
ALIC-L	4.463	8.576	**6.735**	8.487	8.472	8.228
PLIC-R	2.305	3.811	**2.997**	3.653	3.723	3.596
PLIC-L	3.474	7.072	**4.838**	6.901	6.935	6.717
RLIC-R	2.426	4.542	**3.333**	4.326	4.404	4.185
RLIC-L	2.987	4.997	**3.246**	4.721	4.86	4.915
ACR-R	1.691	2.569	2.459	2.448	2.486	**2.224**
ACR-L	2.281	3.736	3.135	3.218	3.299	**3.122**
SCR-R	1.88	3.446	**2.955**	3.407	3.435	3.325
SCR-L	3.617	7.646	**5.211**	7.09	7.162	6.627
PCR-R	1.245	2.995	**2.19**	2.834	2.773	2.77
PCR-L	2.585	5.717	**4.662**	5.296	5.275	4.829
PTR-R	2.027	4.867	**4.293**	4.853	4.811	4.491
PTR-L	3.28	6.902	**4.41**	6.631	6.653	5.997
SS-R	3.136	4.241	4.235	4.211	4.153	**3.819**
SS-L	3.456	5.32	**3.19**	5.222	5.104	5.115
EC-R	1.623	3.248	3.321	3.067	3.084	**2.767**
EC-L	1.887	3.033	**2.406**	2.703	2.728	2.672
CGC-R	2.754	3.682	**2.631**	3.57	3.407	2.868
CGC-L	5.231	8.127	**5.898**	7.777	7.876	6.926
CGH-R	2.048	5.438	**3.64**	5.73	5.296	4.947
CGH-L	3.226	6.181	**3.55**	6.105	6.12	5.991
FXST-R	2.657	4.157	**3.741**	4.341	4.063	4.15
FXST-L	3.382	5.425	**3.347**	5.139	5.044	5.269
SLF-R	1.964	3.701	**2.711**	3.774	3.741	3.648
SLF-L	3.091	6.39	**5.116**	5.97	5.988	5.318
SFO-R	3.287	5.503	**4.388**	5.154	5.249	4.969
SFO-L	5.448	8.469	7.166	7.28	7.704	**7.048**
UNC-R	3.389	5.169	**3.83**	5.121	5.101	4.596
UNC-L	2.135	4.589	**3.704**	4.316	4.249	3.716
TPT-R	2.797	5.378	**3.545**	5.115	4.902	4.959
TPT-L	4.517	10.36	**6.169**	9.479	9.101	8.125

Table S16: Coefficient of variation (%) for AD estimation via no denoising (RAW), P2S, BM4D, MPPCA, and SWIN for white matter regions in HCP test-retest data. Best results are **bolded**.

White Matter Regions	GT	RAW	P2S	MPPCA	BM4D	SWIN
MCP	0.9666	1.73	2.251	1.65	1.684	**1.58**
PCT	1.277	2.307	2.614	2.047	2.024	**1.865**
GCC	2.995	**1.74**	2.762	2.047	1.9	1.909
BCC	1.52	**1.627**	2.767	1.74	1.663	1.678
SCC	1.347	1.386	3.033	**1.381**	1.385	1.381
FX	2.441	3.258	3.824	3.366	3.061	**2.969**
CST-R	1.572	2.09	2.583	2.121	2.048	**1.91**
CST-L	1.254	2.277	2.572	2.225	2.161	**2.001**
ML-R	1.316	2.433	2.625	2.227	2.195	**2.132**
ML-L	1.099	2.586	2.833	2.755	2.544	**2.305**
ICP-R	1.305	2.467	2.728	2.246	2.216	**2.042**

ICP-L	1.313	2.685	2.845	2.484	2.561	**2.271**
SCP-R	1.445	**2.053**	2.959	2.165	2.105	2.157
SCP-L	1.264	2.605	3.584	2.664	2.591	**2.565**
CP-R	1.52	2.264	2.751	2.302	2.259	**2.252**
CP-L	1.718	2.847	3.487	2.819	2.8	**2.673**
ALIC-R	1.588	**1.85**	2.695	2.208	2.008	2.04
ALIC-L	2.83	**3.5**	4.992	3.992	3.678	3.613
PLIC-R	0.7791	1.408	2.29	1.404	1.407	**1.372**
PLIC-L	1.676	2.519	3.912	**2.414**	2.468	2.422
RLIC-R	1.642	2.108	2.91	2.158	2.12	**2.057**
RLIC-L	1.788	1.74	2.772	1.906	1.8	**1.659**
ACR-R	1.779	1.731	2.118	1.822	1.754	**1.693**
ACR-L	2.723	**1.986**	2.662	2.36	2.212	2.219
SCR-R	1.169	2.219	2.855	**2.009**	2.069	2.063
SCR-L	2.231	**2.431**	3.858	2.77	2.688	2.656
PCR-R	0.9502	1.741	2.194	1.73	1.692	**1.666**
PCR-L	1.968	2.654	3.238	2.844	2.707	**2.558**
PTR-R	1.431	1.948	2.856	**1.917**	1.933	1.919
PTR-L	1.754	1.85	3.071	1.867	1.86	**1.719**
SS-R	2.224	**2.712**	3.024	2.876	2.798	2.741
SS-L	1.799	1.881	2.47	2.095	1.94	**1.843**
EC-R	1.399	**2.094**	2.303	2.283	2.175	2.103
EC-L	1.49	**1.586**	1.99	1.794	1.695	1.627
CGC-R	1.594	1.918	2.293	2.058	2.103	**1.915**
CGC-L	2.785	**3.325**	4.139	3.772	3.643	3.587
CGH-R	1.642	3.174	**2.669**	3.538	2.915	3.008
CGH-L	1.858	2.564	2.75	2.927	2.585	**2.349**
FXST-R	1.744	**2.245**	2.352	2.512	2.411	2.298
FXST-L	1.668	**1.999**	2.526	2.314	2.115	2.063
SLF-R	1.165	**1.668**	2.243	1.671	1.693	1.677
SLF-L	2.151	**3.199**	3.882	3.594	3.502	3.368
SFO-R	1.93	2.858	3.737	2.773	2.717	**2.581**
SFO-L	4.139	**5.298**	5.767	5.61	5.57	5.314
UNC-R	1.792	2.965	3.445	2.916	2.922	**2.817**
UNC-L	1.646	3.124	2.891	3.263	3.014	**2.848**
TPT-R	1.721	2.927	3.331	**2.888**	2.979	2.94
TPT-L	2.405	5.428	4.492	4.707	4.251	**3.832**

Table S17: Coefficient of variation (%) for NDI estimation via no denoising (RAW), P2S, BM4D, MPPCA, and SWIN for gray matter cortical regions in HCP test-retest data. Best results are **bolded**.

Cortical Regions	RAW	P2S	MPPCA	BM4D	SWIN
lh-bankssts	4.446	4.584	4.447	4.535	**1.515**
lh-caudalanteriorcingulate	5.382	5.504	5.378	5.56	**2.84**
lh-caudalmiddlefrontal	4.747	4.848	4.746	4.79	**2.041**
lh-cuneus	4.533	4.605	4.532	4.561	**2.044**
lh-entorhinal	5.223	5.491	5.224	5.372	**3.385**
lh-fusiform	4.042	4.234	4.044	4.012	**1.475**
lh-inferiorparietal	5.157	5.151	5.156	5.244	**2.358**
lh-inferiortemporal	4.396	4.599	4.4	4.503	**2.0**
lh-isthmuscingulate	4.489	4.641	4.486	4.577	**2.263**
lh-lateraloccipital	4.753	4.774	4.745	4.748	**2.273**
lh-lateralorbitofrontal	4.031	4.211	4.031	4.141	**2.293**
lh-lingual	4.372	4.501	4.372	4.434	**2.126**
lh-medialorbitofrontal	4.627	4.883	4.629	4.798	**3.064**
lh-middletemporal	5.06	5.265	5.06	5.174	**2.189**
lh-parahippocampal	4.564	4.754	4.564	4.689	**2.094**
lh-paracentral	5.069	5.229	5.069	5.115	**2.376**

lh-parsopercularis	5.303	5.443	5.302	5.267	**2.585**
lh-parsorbitalis	5.418	5.507	5.424	5.481	**3.185**
lh-parstriangularis	5.575	5.709	5.577	5.521	**2.88**
lh-pericalcarine	4.302	4.538	4.301	4.417	**1.686**
lh-postcentral	4.673	4.82	4.672	4.611	**1.989**
lh-posteriorcingulate	4.448	4.694	4.446	4.42	**1.525**
lh-precentral	4.227	4.379	4.227	4.183	**1.626**
lh-precuneus	4.317	4.422	4.317	4.41	**1.483**
lh-rostralanteriorcingulate	5.784	5.874	5.781	6.086	**3.603**
lh-rostralmiddlefrontal	5.911	6.001	5.911	5.762	**3.483**
lh-superiorfrontal	5.729	5.814	5.729	5.604	**3.173**
lh-superiorparietal	4.95	4.934	4.949	4.973	**2.124**
lh-superiortemporal	4.41	4.543	4.41	4.538	**1.777**
lh-supramarginal	4.698	4.799	4.697	4.691	**1.855**
lh-frontalpole	7.348	7.478	7.349	7.381	**6.353**
lh-temporalpole	7.0	7.17	7.005	7.116	**5.154**
lh-transversetemporal	4.717	4.967	4.717	4.797	**2.222**
lh-insula	4.294	4.47	4.293	4.352	**1.646**
rh-bankssts	4.546	4.598	4.548	4.645	**1.639**
rh-caudalanteriorcingulate	4.877	5.033	4.871	4.99	**2.289**
rh-caudalmiddlefrontal	5.39	5.54	5.39	5.4	**2.768**
rh-cuneus	4.158	4.217	4.16	4.152	**1.513**
rh-entorhinal	4.614	4.858	4.613	4.872	**2.556**
rh-fusiform	4.245	4.508	4.253	4.321	**1.766**
rh-inferiorparietal	5.872	5.862	5.873	6.017	**2.965**
rh-inferiortemporal	4.673	4.863	4.665	4.78	**2.499**
rh-isthmuscingulate	3.865	4.023	3.863	3.973	**1.573**
rh-lateraloccipital	4.875	4.951	4.873	4.911	**2.441**
rh-lateralorbitofrontal	4.014	4.165	4.014	4.096	**2.271**
rh-lingual	4.066	4.234	4.066	4.144	**1.859**
rh-medialorbitofrontal	4.602	4.731	4.604	4.738	**3.058**
rh-middletemporal	5.63	5.738	5.63	5.72	**2.681**
rh-parahippocampal	3.981	4.178	3.979	4.186	**1.665**
rh-paracentral	4.842	4.912	4.843	4.911	**2.075**
rh-parsopercularis	5.778	5.789	5.776	5.885	**2.967**
rh-parsorbitalis	5.631	5.63	5.634	5.727	**3.536**
rh-parstriangularis	6.237	6.305	6.236	6.238	**3.567**
rh-pericalcarine	4.166	4.164	4.165	4.144	**1.584**
rh-postcentral	4.792	4.925	4.791	4.801	**2.159**
rh-posteriorcingulate	4.322	4.481	4.323	4.4	**1.363**
rh-precentral	4.968	5.027	4.968	4.95	**2.346**
rh-precuneus	4.275	4.386	4.274	4.309	**1.487**
rh-rostralanteriorcingulate	5.218	5.352	5.221	5.36	**2.992**
rh-rostralmiddlefrontal	6.232	6.35	6.232	6.247	**3.743**
rh-superiorfrontal	5.542	5.643	5.542	5.451	**3.038**
rh-superiorparietal	4.914	4.912	4.915	4.974	**2.078**
rh-superiortemporal	5.326	5.391	5.325	5.432	**2.539**
rh-supramarginal	5.349	5.354	5.349	5.48	**2.508**
rh-frontalpole	6.226	6.288	6.229	6.331	**5.004**
rh-temporalpole	6.107	6.279	6.112	6.309	**4.05**
rh-transversetemporal	4.248	4.383	4.25	4.392	**2.006**
rh-insula	4.567	4.686	4.562	4.655	**1.863**

Table S18: Coefficient of variation (%) for ODI estimation via no denoising (RAW), P2S, BM4D, MPPCA, and SWIN for gray matter cortical regions in HCP test-retest data. Best results are **bolded**.

Cortical Regions	RAW	P2S	MPPCA	BM4D	SWIN
lh-bankssts	1.456	1.435	1.459	1.561	**1.218**

lh-caudalanteriorcingulate	1.894	1.931	1.897	2.087	**1.875**
lh-caudalmiddlefrontal	2.444	**2.436**	2.446	2.651	2.44
lh-cuneus	1.393	1.381	1.393	1.579	**1.286**
lh-entorhinal	1.61	1.622	1.624	1.801	**1.585**
lh-fusiform	1.156	1.175	1.152	1.226	**0.9686**
lh-inferiorparietal	1.503	1.486	1.503	1.599	**1.201**
lh-inferiortemporal	1.514	1.518	1.514	1.627	**1.292**
lh-isthmuscingulate	1.433	**1.382**	1.432	1.675	1.555
lh-lateraloccipital	1.327	1.375	1.331	1.556	**1.288**
lh-lateralorbitofrontal	1.391	**1.343**	1.381	1.57	1.492
lh-lingual	0.9848	0.9468	0.9772	1.184	**0.9448**
lh-medialorbitofrontal	1.629	1.638	**1.594**	1.892	1.73
lh-middletemporal	1.6	1.613	1.6	1.743	**1.391**
lh-parahippocampal	1.638	1.583	1.645	1.712	**1.398**
lh-paracentral	1.383	**1.37**	1.384	1.564	1.546
lh-parsopercularis	**2.046**	2.064	2.048	2.306	2.174
lh-parsorbitalis	2.042	**2.007**	2.051	2.165	2.178
lh-parstriangularis	2.61	**2.574**	2.617	2.858	2.743
lh-pericalcarine	1.554	**1.516**	1.551	1.668	1.562
lh-postcentral	1.338	1.34	**1.335**	1.495	1.421
lh-posteriorcingulate	1.095	1.104	1.091	1.217	**1.068**
lh-precentral	**1.671**	1.687	1.676	1.874	1.782
lh-precuneus	1.052	1.08	1.053	1.242	**1.013**
lh-rostralanteriorcingulate	**2.035**	2.053	2.044	2.447	2.388
lh-rostralmiddlefrontal	3.188	**3.158**	3.194	3.48	3.481
lh-superiorfrontal	2.373	**2.365**	2.373	2.615	2.568
lh-superiorparietal	1.847	1.814	1.848	1.956	**1.636**
lh-superiortemporal	1.419	1.431	1.42	1.465	**1.23**
lh-supramarginal	1.417	1.437	1.419	1.542	**1.309**
lh-frontalpole	3.573	**3.492**	3.587	3.986	3.907
lh-temporalpole	2.376	2.404	2.415	2.693	**2.357**
lh-transversetemporal	1.075	1.135	**1.071**	1.269	1.08
lh-insula	1.848	1.877	1.861	1.923	**1.798**
rh-bankssts	1.439	1.428	1.438	1.575	**1.324**
rh-caudalanteriorcingulate	2.74	**2.704**	2.755	3.085	2.784
rh-caudalmiddlefrontal	2.134	2.115	2.133	2.326	**2.107**
rh-cuneus	1.533	**1.489**	1.536	1.575	1.575
rh-entorhinal	1.513	1.46	1.527	1.705	**1.364**
rh-fusiform	**0.9599**	0.9971	0.9683	1.08	0.9813
rh-inferiorparietal	1.266	1.296	1.266	1.313	**1.118**
rh-inferiortemporal	**1.185**	1.227	1.203	1.391	1.256
rh-isthmuscingulate	1.842	1.799	1.851	1.948	**1.767**
rh-lateraloccipital	**1.084**	1.106	1.085	1.242	1.137
rh-lateralorbitofrontal	1.293	1.282	**1.275**	1.557	1.442
rh-lingual	1.326	**1.324**	1.332	1.486	1.348
rh-medialorbitofrontal	1.497	**1.455**	1.582	1.966	1.836
rh-middletemporal	1.948	1.95	1.942	2.132	**1.806**
rh-parahippocampal	1.194	**1.113**	1.196	1.214	1.155
rh-paracentral	**2.096**	2.112	2.097	2.299	2.204
rh-parsopercularis	2.25	**2.238**	2.256	2.549	2.264
rh-parsorbitalis	2.625	2.624	**2.622**	3.03	2.718
rh-parstriangularis	3.084	3.078	**3.076**	3.486	3.13
rh-pericalcarine	1.342	**1.291**	1.346	1.608	1.477
rh-postcentral	**1.463**	1.489	1.464	1.659	1.527
rh-posteriorcingulate	2.026	**1.996**	2.027	2.258	2.037
rh-precentral	1.692	**1.689**	1.69	1.831	1.714
rh-precuneus	1.609	1.588	1.611	1.619	**1.49**
rh-rostralanteriorcingulate	**2.596**	2.605	2.615	2.984	2.936
rh-rostralmiddlefrontal	3.121	**3.084**	3.124	3.512	3.17

rh-superiorfrontal	2.776	**2.771**	2.778	3.066	2.927
rh-superiorparietal	**1.175**	1.242	1.175	1.334	1.208
rh-superiortemporal	1.671	1.677	1.665	1.896	**1.588**
rh-supramarginal	1.399	1.446	1.4	1.488	**1.253**
rh-frontalpole	2.726	**2.707**	2.712	3.12	3.005
rh-temporalpole	**2.084**	2.117	2.154	2.544	2.156
rh-transversetemporal	1.567	**1.552**	1.567	1.891	1.661
rh-insula	1.566	1.543	1.566	1.77	**1.481**

Table S19: Coefficient of variation (%) for FISO estimation via no denoising (RAW), P2S, BM4D, MPPCA, and SWIN for gray matter cortical regions in HCP test-retest data. Best results are **bolded**.

Cortical Regions	RAW	P2S	MPPCA	BM4D	SWIN
lh-bankssts	20.25	20.62	20.26	22.03	**16.25**
lh-caudalanteriorcingulate	16.0	15.88	15.99	16.66	**14.27**
lh-caudalmiddlefrontal	15.85	16.57	15.86	16.71	**12.2**
lh-cuneus	12.74	13.56	12.74	13.57	**9.165**
lh-entorhinal	13.39	13.91	13.39	13.88	**10.67**
lh-fusiform	15.35	16.14	15.35	16.23	**10.5**
lh-inferiorparietal	14.08	14.7	14.09	15.04	**10.32**
lh-inferiortemporal	17.69	18.86	17.69	18.62	**13.32**
lh-isthmuscingulate	11.52	12.06	11.52	12.05	**9.708**
lh-lateraloccipital	11.84	12.4	11.84	12.08	**6.857**
lh-lateralorbitofrontal	11.94	13.39	11.94	12.86	**8.725**
lh-lingual	9.904	10.48	9.905	10.36	**6.796**
lh-medialorbitofrontal	13.97	14.12	13.97	14.94	**11.49**
lh-middletemporal	17.8	17.49	17.81	18.85	**13.93**
lh-parahippocampal	14.86	15.2	14.86	15.54	**10.94**
lh-paracentral	15.14	15.31	15.14	15.55	**10.45**
lh-parsopercularis	15.18	15.57	15.19	16.31	**11.26**
lh-parsorbitalis	17.53	17.62	17.53	19.02	**13.48**
lh-parstriangularis	17.03	17.19	17.03	18.7	**13.15**
lh-pericalcarine	12.32	13.36	12.31	13.33	**8.79**
lh-postcentral	13.57	13.87	13.57	14.16	**9.935**
lh-posteriorcingulate	13.8	14.19	13.79	14.74	**9.548**
lh-precentral	14.45	14.91	14.45	15.08	**10.56**
lh-precuneus	14.64	15.21	14.64	15.18	**9.929**
lh-rostralanteriorcingulate	15.69	16.16	15.7	16.46	**12.45**
lh-rostralmiddlefrontal	14.31	14.3	14.31	14.89	**10.59**
lh-superiorfrontal	13.24	13.78	13.23	13.42	**9.182**
lh-superiorparietal	14.43	15.14	14.43	14.68	**9.181**
lh-superiortemporal	11.94	12.29	11.95	12.1	**7.953**
lh-supramarginal	15.02	15.2	15.02	15.65	**10.54**
lh-frontalpole	14.73	14.31	14.74	15.14	**12.12**
lh-temporalpole	20.92	20.67	20.92	22.12	**18.51**
lh-transversetemporal	12.84	12.87	12.84	13.42	**9.818**
lh-insula	14.09	14.23	14.1	15.13	**10.31**
rh-bankssts	17.14	17.59	17.15	17.86	**11.53**
rh-caudalanteriorcingulate	15.39	15.8	15.38	16.43	**11.81**
rh-caudalmiddlefrontal	17.9	18.35	17.9	18.33	**12.69**
rh-cuneus	13.66	14.18	13.66	14.69	**10.41**
rh-entorhinal	12.66	13.45	12.67	13.51	**9.524**
rh-fusiform	15.53	17.02	15.53	16.22	**11.71**
rh-inferiorparietal	20.28	20.66	20.28	21.22	**16.56**
rh-inferiortemporal	17.04	18.11	17.04	17.74	**13.74**
rh-isthmuscingulate	13.85	14.19	13.87	15.09	**9.837**
rh-lateraloccipital	16.01	16.75	16.0	16.64	**12.49**
rh-lateralorbitofrontal	13.18	13.91	13.18	14.22	**10.8**

rh-lingual	12.02	12.26	12.03	12.63	**7.996**
rh-medialorbitofrontal	14.0	14.7	14.01	14.7	**10.17**
rh-middletemporal	20.97	20.84	20.96	22.1	**17.85**
rh-parahippocampal	14.12	14.35	14.12	14.94	**9.904**
rh-paracentral	16.19	16.31	16.19	16.94	**11.87**
rh-parsopercularis	14.7	14.71	14.7	15.55	**10.51**
rh-parsorbitalis	13.52	12.93	13.52	14.24	**12.31**
rh-parstriangularis	16.5	16.46	16.5	17.15	**13.15**
rh-pericalcarine	12.98	13.23	12.98	13.78	**8.894**
rh-postcentral	12.71	13.27	12.71	13.08	**8.309**
rh-posteriorcingulate	16.29	16.5	16.29	17.59	**11.32**
rh-precentral	15.58	16.4	15.58	15.89	**10.88**
rh-precuneus	14.46	14.78	14.46	15.13	**10.06**
rh-rostralanteriorcingulate	15.47	15.37	15.46	15.8	**11.04**
rh-rostralmiddlefrontal	15.84	15.72	15.84	16.55	**11.49**
rh-superiorfrontal	13.28	13.9	13.28	13.53	**9.48**
rh-superiorparietal	15.21	15.76	15.21	15.48	**9.97**
rh-superiortemporal	16.04	16.31	16.03	16.7	**11.89**
rh-supramarginal	18.14	18.57	18.13	18.79	**13.99**
rh-frontalpole	13.36	12.74	13.36	13.82	**10.89**
rh-temporalpole	15.42	15.98	15.41	16.28	**11.37**
rh-transversetemporal	14.0	14.7	14.01	14.66	**11.29**
rh-insula	11.83	11.91	11.84	12.2	**8.222**

Table S20: Coefficient of variation (%) for NDI estimation via no denoising (RAW), P2S, BM4D, MPPCA, and SWIN for white matter regions in HCP test-retest data. Best results are **bolded**.

White Matter Regions	RAW	P2S	MPPCA	BM4D	SWIN
MCP	1.515	1.779	1.515	1.581	**1.008**
PCT	2.165	2.413	2.168	2.454	**1.856**
GCC	2.297	2.574	2.297	2.461	**2.092**
BCC	2.411	2.632	2.408	2.41	**1.355**
SCC	1.662	1.935	1.66	1.663	**0.9457**
FX	3.456	3.894	3.456	3.618	**2.446**
CST-R	1.727	2.007	1.728	1.928	**1.41**
CST-L	1.695	1.975	1.696	1.906	**1.313**
ML-R	2.806	3.067	2.803	2.915	**2.012**
ML-L	2.74	3.079	2.742	2.953	**1.986**
ICP-R	2.492	2.715	2.493	2.44	**1.568**
ICP-L	2.175	2.4	2.176	2.192	**1.213**
SCP-R	2.055	2.312	2.053	2.137	**1.292**
SCP-L	2.101	2.317	2.099	2.271	**1.278**
CP-R	1.494	1.654	1.492	1.627	**1.193**
CP-L	1.567	1.708	1.567	1.637	**1.166**
ALIC-R	1.962	2.357	1.962	2.149	**1.29**
ALIC-L	2.795	3.181	2.795	3.029	**2.27**
PLIC-R	1.697	1.926	1.696	1.757	**1.058**
PLIC-L	2.386	2.575	2.389	2.575	**1.844**
RLIC-R	2.673	2.928	2.671	2.746	**1.789**
RLIC-L	2.416	2.737	2.417	2.468	**1.508**
ACR-R	2.871	3.233	2.874	3.019	**2.289**
ACR-L	3.576	3.879	3.575	3.685	**3.245**
SCR-R	2.336	2.615	2.333	2.276	**1.434**
SCR-L	2.563	2.655	2.561	2.522	**1.342**
PCR-R	2.463	2.769	2.461	2.421	**1.107**
PCR-L	2.652	2.753	2.652	2.578	**1.205**
PTR-R	2.754	3.038	2.75	2.741	**1.5**
PTR-L	2.74	2.961	2.737	2.819	**1.297**

SS-R	3.261	3.464	3.263	3.38	**2.311**
SS-L	2.9	3.141	2.898	2.937	**1.813**
EC-R	3.198	3.587	3.189	3.265	**1.631**
EC-L	3.293	3.563	3.284	3.362	**1.985**
CGC-R	2.458	2.679	2.458	2.581	**1.472**
CGC-L	2.676	3.017	2.677	2.731	**1.678**
CGH-R	2.735	3.183	2.73	2.843	**1.826**
CGH-L	2.928	3.319	2.931	3.131	**2.025**
FXST-R	2.734	3.012	2.73	2.913	**1.8**
FXST-L	2.612	2.935	2.608	2.758	**1.613**
SLF-R	2.215	2.49	2.215	2.154	**1.237**
SLF-L	2.29	2.477	2.289	2.353	**1.227**
SFO-R	2.4	2.858	2.392	2.476	**1.663**
SFO-L	4.2	4.516	4.2	4.327	**3.189**
UNC-R	4.15	4.42	4.147	4.146	**2.478**
UNC-L	3.321	3.598	3.326	3.437	**1.898**
TPT-R	3.138	3.199	3.143	3.21	**1.465**
TPT-L	3.327	3.476	3.328	3.461	**2.015**

Table S21: Coefficient of variation (%) for ODI estimation via no denoising (RAW), P2S, BM4D, MPPCA, and SWIN for white matter regions in HCP test-retest data. Best results are **bolded**.

White Matter Regions	RAW	P2S	MPPCA	BM4D	SWIN
MCP	3.362	3.657	3.388	3.313	**3.021**
PCT	6.411	7.023	6.45	6.495	**5.759**
GCC	4.48	8.522	4.374	**4.372**	4.732
BCC	2.954	6.584	**2.948**	3.064	3.194
SCC	2.629	6.068	**2.62**	2.708	3.016
FX	4.123	6.529	4.097	3.882	**3.714**
CST-R	**5.267**	7.198	5.27	5.845	5.718
CST-L	**6.191**	8.277	6.199	6.811	6.217
ML-R	5.546	7.705	5.553	5.584	**5.075**
ML-L	4.503	7.564	**4.494**	4.728	4.665
ICP-R	4.943	4.548	4.97	4.836	**3.78**
ICP-L	4.963	4.89	4.954	5.272	**4.088**
SCP-R	4.466	5.366	4.499	4.665	**4.013**
SCP-L	**3.902**	5.28	3.914	4.248	4.151
CP-R	3.911	5.433	**3.901**	4.143	4.416
CP-L	4.836	5.506	4.855	5.681	**4.735**
ALIC-R	**5.263**	9.024	5.267	5.578	5.486
ALIC-L	**6.078**	8.724	6.107	6.588	6.101
PLIC-R	**3.888**	5.312	3.888	4.088	4.374
PLIC-L	4.902	5.99	4.888	5.133	**4.598**
RLIC-R	**5.925**	7.585	5.95	6.164	5.933
RLIC-L	5.959	7.082	6.006	6.329	**5.658**
ACR-R	4.905	4.691	5.113	5.115	**4.483**
ACR-L	5.189	5.716	5.338	5.384	**5.077**
SCR-R	3.897	3.999	3.94	4.041	**3.087**
SCR-L	4.123	3.892	4.131	4.213	**3.403**
PCR-R	5.427	5.845	5.414	5.454	**4.752**
PCR-L	4.327	4.51	4.324	4.448	**3.751**
PTR-R	3.826	7.196	3.81	3.832	**3.388**
PTR-L	3.607	6.62	3.624	3.528	**3.126**
SS-R	5.301	6.409	5.387	5.699	**4.462**
SS-L	4.796	7.46	4.807	5.135	**4.596**
EC-R	4.632	4.821	4.794	5.079	**3.874**
EC-L	5.111	5.141	5.238	5.609	**4.201**
CGC-R	6.461	11.34	6.417	**5.946**	6.844

CGC-L	**4.02**	7.941	4.034	4.17	4.294
CGH-R	**6.96**	11.87	6.989	8.189	8.631
CGH-L	**8.883**	14.75	8.905	11.35	11.26
FXST-R	7.167	9.159	7.218	7.952	**6.859**
FXST-L	**5.483**	9.262	5.524	6.995	5.909
SLF-R	4.322	6.852	4.352	4.417	**4.234**
SLF-L	4.154	6.107	4.185	4.239	**3.653**
SFO-R	6.589	4.726	6.671	6.987	**4.415**
SFO-L	7.992	**6.05**	8.138	8.054	6.105
UNC-R	10.94	9.57	11.27	11.64	**7.213**
UNC-L	9.61	9.845	9.77	9.422	**8.762**
TPT-R	6.067	7.771	6.078	5.889	**4.38**
TPT-L	8.446	**7.238**	8.596	7.308	8.824

Table S22: Coefficient of variation (%) for FISO estimation via no denoising (RAW), P2S, BM4D, MPPCA, and SWIN for white matter regions in HCP test-retest data. Best results are **bolded**.

White Matter Regions	RAW	P2S	MPPCA	BM4D	SWIN
MCP	9.293	11.18	9.293	9.485	**8.199**
PCT	10.05	11.01	10.05	10.69	**9.466**
GCC	8.581	9.76	8.595	8.68	**7.502**
BCC	13.03	14.86	13.04	14.01	**11.4**
SCC	14.05	15.38	14.04	14.56	**13.58**
FX	10.7	11.71	10.7	11.24	**9.04**
CST-R	7.849	9.142	7.858	8.213	**7.474**
CST-L	7.481	8.844	7.479	7.854	**7.359**
ML-R	14.83	**14.73**	14.83	15.57	15.43
ML-L	**15.23**	15.5	15.24	16.03	15.48
ICP-R	13.72	15.04	13.72	14.3	**12.28**
ICP-L	12.23	13.42	12.23	12.89	**11.13**
SCP-R	6.914	7.976	6.916	6.785	**6.027**
SCP-L	9.039	9.853	9.041	8.903	**8.5**
CP-R	12.29	**12.16**	12.29	12.95	12.56
CP-L	11.49	**11.24**	11.49	11.8	12.57
ALIC-R	11.26	12.56	11.27	11.73	**10.45**
ALIC-L	**27.1**	29.05	27.14	30.64	27.98
PLIC-R	7.903	9.073	7.91	8.285	**6.797**
PLIC-L	22.36	**21.75**	22.38	24.67	22.52
RLIC-R	12.57	13.76	12.56	13.09	**10.55**
RLIC-L	16.96	16.85	16.94	17.86	**15.65**
ACR-R	10.04	9.664	10.06	10.68	**8.19**
ACR-L	11.13	11.61	11.17	11.95	**9.5**
SCR-R	18.75	18.7	18.76	19.83	**17.05**
SCR-L	27.49	**25.22**	27.52	30.04	27.06
PCR-R	16.25	16.56	16.25	16.88	**14.29**
PCR-L	24.65	22.98	24.64	26.66	**22.13**
PTR-R	13.36	14.61	13.35	14.09	**11.48**
PTR-L	19.24	21.29	19.24	20.65	**19.07**
SS-R	15.01	15.48	15.02	16.12	**13.1**
SS-L	13.72	15.49	13.73	14.52	**12.85**
EC-R	18.27	18.17	18.25	20.9	**14.93**
EC-L	18.64	19.23	18.65	21.78	**16.17**
CGC-R	9.315	11.29	9.309	9.842	**7.469**
CGC-L	16.24	17.55	16.23	16.62	**15.79**
CGH-R	10.94	11.69	10.93	11.8	**8.834**
CGH-L	14.5	15.3	14.51	16.55	**13.36**
FXST-R	10.72	10.82	10.72	11.39	**9.431**
FXST-L	14.11	14.27	14.11	14.27	**12.34**

SLF-R	13.89	15.44	13.89	14.49	**11.89**
SLF-L	28.13	28.38	28.13	30.91	**28.06**
SFO-R	16.56	16.77	**16.53**	18.42	17.01
SFO-L	31.93	**30.03**	31.85	36.08	32.12
UNC-R	32.87	**29.24**	32.94	40.59	31.43
UNC-L	22.72	21.77	22.79	27.0	**17.55**
TPT-R	13.09	12.68	13.08	13.26	**12.1**
TPT-L	29.37	27.04	29.34	28.02	**26.44**
